# Tudor-SN Interacts with Piwi Antagonistically in Regulating Spermatogenesis but Synergistically in Silencing Transposons in *Drosophila*

**DOI:** 10.1371/journal.pgen.1005813

**Published:** 2016-01-25

**Authors:** Hsueh-Yen Ku, Vamsi K. Gangaraju, Hongying Qi, Na Liu, Haifan Lin

**Affiliations:** Yale Stem Cell Center and Department of Cell Biology, Yale University School of Medicine, New Haven, Connecticut, United States of America; University of California Riverside, UNITED STATES

## Abstract

Piwi proteins associate with piRNAs and functions in epigenetic programming, post-transcriptional regulation, transposon silencing, and germline development. However, it is not known whether the diverse functions of these proteins are molecularly separable. Here we report that Piwi interacts with Tudor-SN (Tudor staphylococcal nuclease, TSN) antagonistically in regulating spermatogenesis but synergistically in silencing transposons. However, it is not required for piRNA biogenesis. TSN is known to participate in diverse molecular functions such as RNAi, degradation of hyper-edited miRNAs, and spliceosome assembly. We show that TSN colocalizes with Piwi in primordial germ cells (PGCs) and embryonic somatic cells. In adult ovaries and testes, TSN is ubiquitously expressed and enriched in the cytoplasm of both germline and somatic cells. The *tsn* mutants display a higher mitotic index of spermatogonia, accumulation of spermatocytes, defects in meiotic cytokinesis, a decreased number of spermatids, and eventually reduced male fertility. Germline-specific TSN-expression analysis demonstrates that this function is germline-dependent. Different from other known Piwi interters, TSN represses Piwi expression at both protein and mRNA levels. Furthermore, reducing *piwi* expression in the germline rescues *tsn* mutant phenotype in a dosage-dependent manner, demonstrating that Piwi and TSN interact antagonistically in germ cells to regulate spermatogenesis. However, the *tsn* deficiency has little, if any, impact on piRNA biogenesis but displays a synergistic effect with *piwi* mutants in transposon de-silencing. Our results reveal the biological function of TSN and its contrasting modes of interaction with Piwi in spermatogenesis, transposon silencing, and piRNA biogenesis.

## Author Summary

Piwi proteins bind to a large class of small noncoding RNAs called Piwi-interacting RNAs (piRNAs). These proteins have emerged as major players in germline development, stem cell self-renewal, transposon silencing, and gene regulation. However, it is not known whether these functions of Piwi proteins represent separate molecular mechanisms. Furthermore, although multiple Piwi interactors have been identified, including Tudor-domain-containing proteins, none of them regulates Piwi expression or interacts with Piwi antagonistically, or only impact on a subset of Piwi functions. Here we show that *Drosophila* Piwi interacts with a special Tudor-domain-containing protein called Tudor-SN (Tudor staphylococcal nuclease, TSN). TSN is drastically different from the known Piwi interactors because it represses Piwi mRNA and protein expression and interacts with Piwi antagonistically in spermatogenesis but synergistically in transposon silencing. However, this interaction is not required for piRNA biogenesis. Our study represents the first demonstration that different functions of Piwi are mediated by different molecular mechanisms. In addition, this is the first *in vivo* study that reveals the biological function of TSN protein in an organism.

## Introduction

PIWI proteins are a subfamily of the PIWI/ARGONAUTE protein family. Piwi proteins associate with Piwi-interacting RNAs (piRNAs) and function in germline stem cell (GSC) self-renewal, germline development, epigenetic programming, post-transcriptional regulation, and transposon silencing [1–3]. The defining member of the Piwi/AGONAUTE family is the Piwi protein in *Drosophila* (PIWI herein stands for the subfamily whereas Piwi specifically stands for the *Drosophila* Piwi protein), which is known to regulate GSC maintenance, germ cell proliferation, heterochromatin formation, and transposon silencing [4–8]. However, it is not known whether the diverse functions of these proteins are molecularly separable; nor it is known whether all Piwi functions are piRNA-dependent. Furthermore, although Piwi proteins are known to interact with multiple proteins, including Tudor-domain-containing proteins, no interactor is known to regulate Piwi expression or interacts with Piwi antagonistically, or only impact on only a subset of Piwi functions.

Here we report that Tudor-SN (Tudor staphylococcal nuclease, TSN), a member of the evolutionarily conserved Tudor protein family, as a novel and unique Piwi-interactor in *Drosophila*. TSN contains five staphylococcal nuclease-like domains (SN1-SN5) and a methyl lysine/arginine recognizing Tudor domain [9–11]. TSN is highly conserved during evolution from fission yeast to mammals, and it exists as a single gene in a species with no close homologue [12]. TSN has been reported to participate in a variety of molecular functions, such as transcriptional activation through interacting with STATs, formation of RNA-induced silencing complex (RISC) and stress granules, degradation of A-to-I hyper-edited miRNAs, and assembly of spliceosome [13–19]. In mice and humans, TSN (a.k.a. SND1) has been demonstrated to interact with mouse and human PIWI proteins MIWI and MILI via binding to the symmetrically dimethylated arginine (sDMA) resides in the PIWI proteins [20, 21], similar to other Tudor family proteins [20, 21]. In *Drosophila*, although Tudor protein family members, including PAPI (Partner of PIWIs) have been shown to interact with PIWI proteins via sDMA residues [22], TSN has not been demonstrated to interact with PIWI proteins. Moreover, the biological role of TSN has not been explored in any organism.

Here we report the biological function of TSN in *Drosophila*, its interaction with Piwi, and the regulatory effect of such interaction. We show that TSN is highly expressed in embryos and adult gonads. In testes, *tsn* mutations result in abnormal spermatogenesis, including a higher mitotic index of spermatogonia, drastically increased number of spermatocytes, defects in meiotic cytokinesis, a reduction in spermatids, and consequently a decline in male fertility. Furthermore, the phenotype of *tsn* mutants is rescued by the mutations of *piwi*, indicating an antagonistic relationship of TSN and Piwi in the germline during spermatogenesis. In support of this, we show that TSN down-regulates the expression of Piwi at both protein and RNA levels. Finally, we demonstrate that *tsn* mutants display little impact on the piRNA biogenesis but have synergistic impact with Piwi on transposon repression. Our data suggest that TSN negatively regulates *piwi* expression in germline development while it may work with the Piwi protein in piRNA biogenesis and transposon silencing.

## Results

### TSN is a novel Piwi interactor

**Fig 1:**
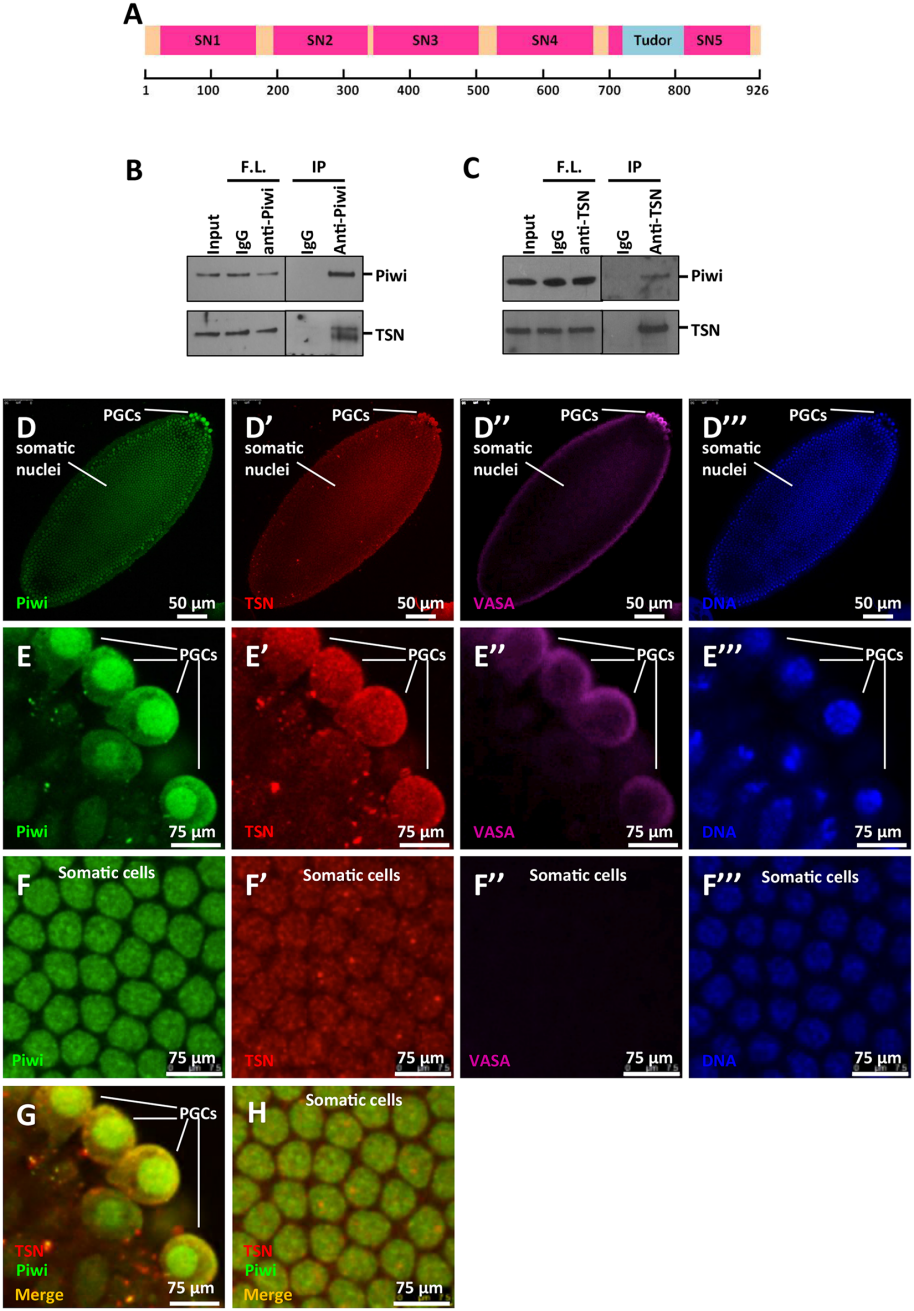
TSN is a novel Piwi-interacting protein. (**A**) Schematic depiction of protein domain structures of TSN. TSN contains five staphylococcal nuclease-like domains (SN1-SN5) and a methyl lysine/arginine recognizing Tudor domain. (**B**) Co-immunoprecipitations of Piwi and TSN with anti-Piwi antibody. Input was the cytoplasmic fraction of the lysates from 0-12h WT embryos. 5% of input was used in the Western blot analysis. IgG was used as the control. F.L., flow-through; IP, immunoprecipitates. (**C**) Reciprocal co-immunoprecipitations of TSN and Piwi with mouse anti-TSN antibody. Input was the cytoplasmic fraction of lysates from 0–12 h WT embryos. 10% of input was used in the Western blot analysis. IgG was used as controls for the co-immunoprecipitations. F.L., flow-through; IP, immunoprecipitates. (**D-F‴**) Immunostaining of Piwi (green), TSN (using rabbit anti-TSN-N antibody, red), and the germ cell marker VASA (purple) in 0-1h WT embryos. DNA was labeled by DAPI (blue). (**D-D‴**) The whole embryo. PGCs, primordial germ cells. (**E-E‴**) Magnified images of E-E‴ showing the region of PGCs. Piwi and TSN were both highly enriched in the cytoplasmic region of PGCs. (**F-F‴**) Magnified images of D-D‴ showing the somatic cells. Piwi and TSN were both localized to the somatic nuclei. **(G)** Co-immunostaining of Piwi and TSN in PGCs, merged image of E and E’. **(H)** Co-immunostaining of Piwi and TSN in somatic cells, merged image of F and F’.

In an attempt to identify novel molecular interactors of Piwi, we previously reported the fractionation of cytoplasmic extracts of 0–12 h *w*^*1118*^ wild-type embryos using size-exclusion chromatography [23]. After the final chromatography column, Piwi migrated with an apparent molecular weight of ~150 kDa. We resolved the peak fraction for Piwi obtained from Superdex 200 chromatography on a 7.5% SDS polyacrylamide gel and stained it with silver stain. We obtained the identities of individual bands by excising bands from a gel stained with colloidal coomassie blue, followed by mass spectrometry [23]. Piwi-Hsp90 interaction was described in our earlier paper [23]. Here we report that TSN, an evolutionarily conserved Tudor protein family member, is another novel Piwi-interacting protein. TSN contains five staphylococcal nuclease-like domains (SN1-SN5) and a methyl lysine/arginine-recognizing Tudor domain (Fig 1A). To confirm Piwi and TSN interaction, we performed co-immunoprecipitation assays using 0–12 h wild-type embryos. Piwi was immunoprecipitated with TSN, and the interaction was further confirmed by reciprocal co-immunoprecipitations, suggesting that Piwi and TSN form a complex *in vivo* (Fig 1B and 1C and S1 Fig). Moreover, immunofluorescence microscopy in 0–2 h wild-type embryos revealed that Piwi is co-localized with TSN in the cytoplasm and nuclei of PGCs as well as in the nuclei of somatic cells (Fig 1D–1H). Taken together, our results suggest that Piwi and TSN physically interact during embryogenesis.

### TSN expression during development

**Fig 2:**
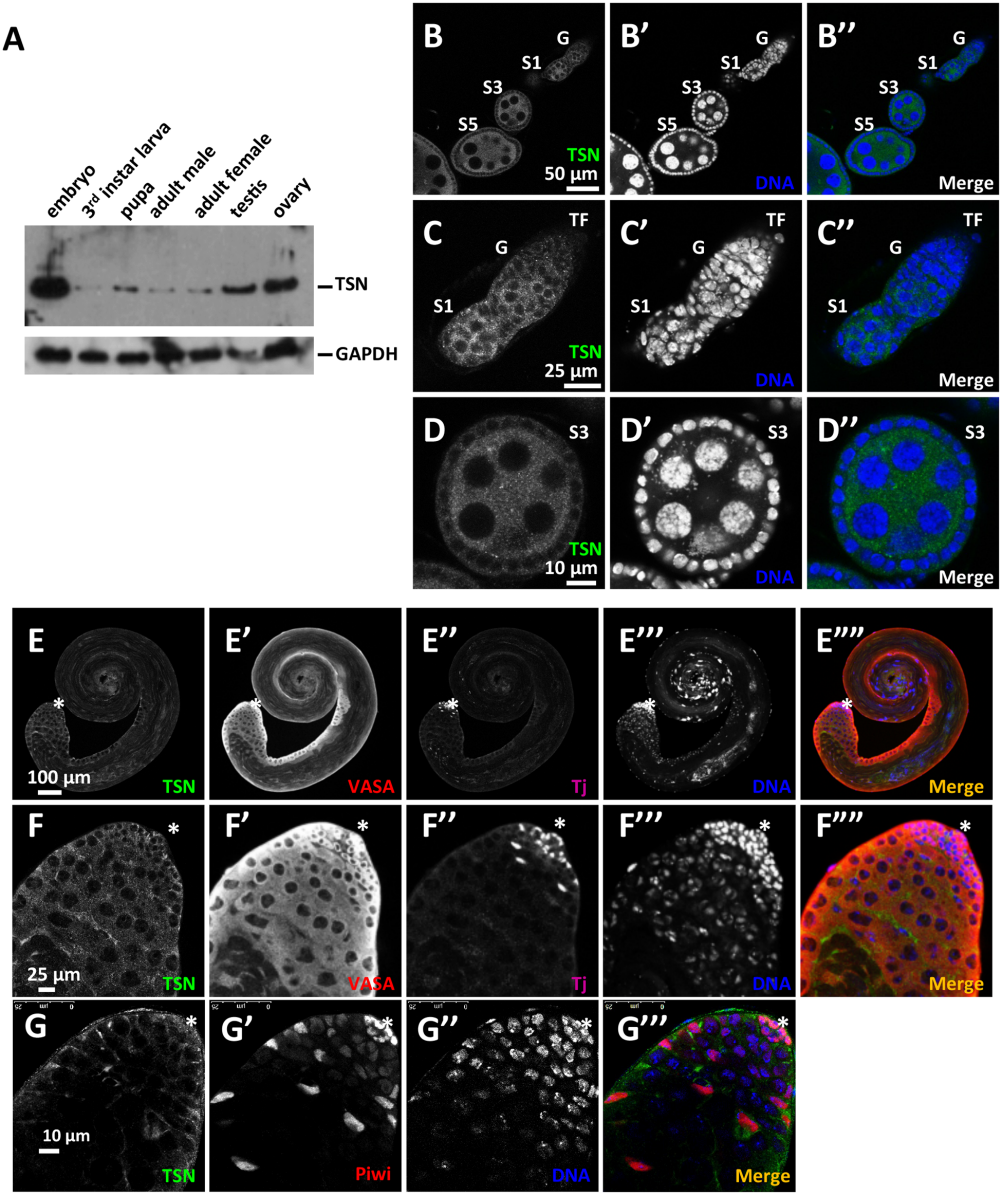
TSN is highly expressed in embryos and adult gonads. (**A**) Expression pattern of TSN protein in different key developmental stages and tissues. Western blot analysis using mouse anti-TSN antibody revealed that TSN was highly expressed in the embryonic stage as well as adult ovaries and testes. (**B-D”**) Immunostaining of TSN (using mouse anti-TSN antibody, green) in adult WT ovaries. DNA was labeled by DAPI (blue). TSN was localized to the cytoplasm of both germline and somatic cells in adult ovaries. G, germarium; S1-S5, stage 1–5 egg chambers, respectively; TF, terminal filament. (**C-C”**) Magnified images of a germarium including a S1 egg chamber. (**D-D”**) Magnified images of B-B” showing the S3 egg chamber. (**E-F””**) Immunostaining TSN (using mouse anti-TSN antibody, green) in WT adult testes. Germ cells were labeled with anti-VASA antibody (red) and somatic cells were labeled with anti-Tj antibody (purple). DNA was labeled by DAPI (blue). TSN was localized to the cytoplasm of germline and somatic cells in adult testes. Magnified images of the apical region of the testis are shown in F-F””. (G-G‴) Immunostaining of TSN (green) and Piwi (red) in WT adult testes. DNA was labeled by DAPI (blue). Piwi was localized in the nuclei of hub cells, early germ cells and somatic cyst cells. Asterisk: the hub.

To investigate the biological function of TSN, we first analyzed the expression of TSN in different tissues and at key stages of development. Western blot analysis indicated that TSN is widely expressed in most developmental stages with the highest expression levels in embryos, adult ovaries and testes (Fig 2A). In addition, we used immunofluorescence microscopy to examine the expression pattern and subcellular localization of TSN in wild-type adult ovaries and testes. TSN is expressed in both the germline and somatic cells, from the germarium to advanced stage egg chambers (Fig 2B–2B”), where TSN is mainly present in the cytoplasm (Fig 2C–2D”). TSN is also expressed throughout spermatogenesis (Fig 2E) in both germ cells (labeled by VASA staining in Fig 2E’) and somatic cells (labeled by Tj staining in Fig 2E”), where TSN was also highly enriched in the cytoplasm (Fig 2F–2F”” and 2G–2G‴). As previously reported [5], Piwi is expressed in the nuclei of hub cells, early germ cells and somatic cyst cells (Fig 2G’ and 2G‴).

### TSN is required in the germline for proper spermatogenesis

**Fig 3:**
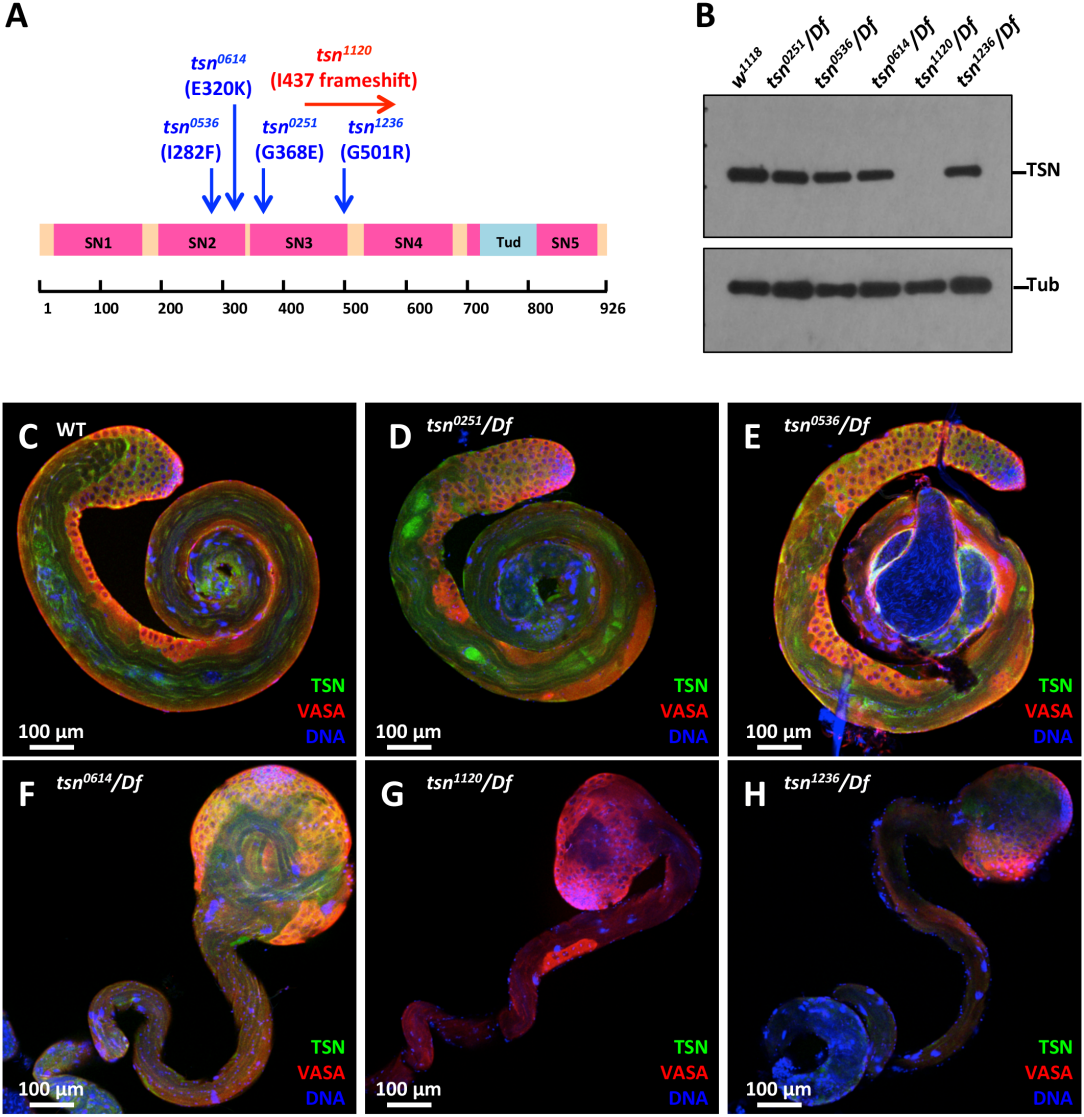
Mutations of *tsn* result in abnormal spermatogenesis. (**A**) The EMS mutant alleles of *tsn*. The position of each mutation is indicated by an arrow above a schematic representation of the TSN protein. *tsn*^*0536*^ and *tsn*^*0614*^ are point mutations at 282Ile→Phe and 320Glu→Lys of the SN2 domain, respectively. *tsn*^*0251*^ and *tsn*^*1236*^ are point mutations at 368Gly→Glu and 501Gly→Arg of the SN3 domain, respectively. *tsn*^*1120*^ is a deletion mutation which causes frameshift from 437Ile of the SN3 domain. (**B**) TSN protein expression level in the adult testes of each mutant allele heterozygous with a deficiency *(Df)* that disrupts the TSN genomic locus was analyzed by Western blots. TSN was undetectable in the lysates from *tsn*^*1120*^/*Df* testes. (**C-H**) Immunostaining of TSN (using mouse anti-TSN antibody, green) and VASA (red) in the testes from the five *tsn* mutant alleles heterozygous with the *Df* allele. DNA was labeled by DAPI (blue). *tsn*^*0614*^/*Df* (F), *tsn*^*1120*^/*Df* (G), and *tsn*^*1236*^/*Df* (H) resulted in the swollen apical tip of the testis. TSN was barely detectable in the *tsn*^*1120*^/*Df* testis (G). *tsn*^*0251*^/*Df* (D) and *tsn*^*0536*^/*Df* (E) did not cause obvious testicular phenotype compared to the WT testis (C).

To determine the biological role of TSN, we first examined five ethyl-methane sulfonate (EMS) mutant alleles of TSN obtained from the Fly TILLING [24] for their molecular lesions. The *tsn*^*0536*^ and *tsn*^*0614*^ are point mutations (282Ile→Phe and 320 Glu→ Lys, respectively) in the SN2 domain. The *tsn*^*0251*^ and *tsn*^*1236*^ are point mutations (368Gly→Glu and 501Gly→Arg, respectively) in the SN3 domain. The *tsn*^*1120*^ mutation is a deletion from +3009(A) to +3067(G) (per FlyBase coordinate of CG7008) that causes a frame shift from 437Ile on (Fig 3A). To determine whether *tsn* mutant alleles affect protein stability, we analyzed the protein level of TSN using the testicular lysates from each mutant allele transheterozygous to a deficiency (*Df*) allele uncovering the *tsn* genomic region. We found that TSN protein levels were undetectable in *tsn* ^*1120*^*/Df* mutants (Fig 3B). Moreover, antibodies recognizing epitopes in the N- or C-terminal region of TSN failed to recognize detectable protein levels in *tsn* ^*1120*^*/Df* testes, indicating that *tsn* ^*1120*^ is a protein-null allele.

To further examine the role of TSN in spermatogenesis, we performed immunostaining of 2-day-old mutant testes. Testes in *tsn*^*0251*^*/Df* and *tsn*^*0536*^*/Df* mutants (Fig 3D and 3E) were similar to *tsn*^+^*/tsn*^+^ control testes (Fig 3C), suggesting these mutations fail to disrupt TSN function. However, *tsn*^*0614*^*/Df*, *tsn* ^*1120*^*/Df*, and *tsn*^*1236*^*/Df* alleles resulted in the swollen apical tips of testes containing an excessive number of early spermatogenic cells, with 42% (n = 84), 86% (n = 81), and 36% (n = 70) of the testes displaying this phenotype, respectively (Fig 3F–3H). Notably, TSN immunostaining signal was undetectable in *tsn* ^*1120*^*/Df* testes (Fig 3G), consistent with the result of our Western blot analysis (Fig 3B). In addition, we analyzed 2-day-old *tsn* ^*1120*^*/tsn*^*0614*^, *tsn* ^*1120*^*/tsn* ^*1236*^, and *tsn* ^*0614*^*/tsn*^*1236*^ transheterozygous males. Interestingly, testes from all of the three transheterozygous mutants exhibited the swollen apical tips, with 81% (n = 80), 78% (n = 79), and 45% (n = 71) of the testes displaying the phenotype, respectively. The knockdown of TSN using *Actin5c-Gal4* driver (S2 Fig) also showed phenotype similar to *tsn*^*1120*^*/tsn*^*0614*^ transheterozygous mutant, though at a lower frequency (36%, n = 42) (S2 Fig). The lower penetrance (compared to that of *tsn* ^*1120*^*/tsn* ^*0614*^ transheterozygous mutant) may due to the modest knockdown efficiency of TSN in testes (S2 Fig). Although the knockdown efficiency of TSN in ovaries is much stronger, we did not observe any obvious defects in TSN knockdown ovaries (S3 Fig) or *tsn*^*1120*^*/tsn*^*0614*^ transheterozygous mutant ovaries (S4C Fig). Together, these data indicate that *tsn* is required for normal progression of spermatogenesis but not oogenesis.

To determine whether the function of *tsn* in spermatogenesis is germline-dependent, we expressed wild-type TSN in germ cells using *nosVP16-Gal4* driver (see Materials and Methods). This expression rescued the swollen testis apical tip phenotype of 68% of the *tsn*^*1120*^*/tsn*^*0614*^ transheterozygous mutants to WT-like (n = 62; S5A Fig), indicating that the *tsn* function in spermatogeneis is germline-dependent.

### *tsn* mutant testes display over-proliferation of spermatogonia and compromised differentiation of spermatocytes

**Fig 4:**
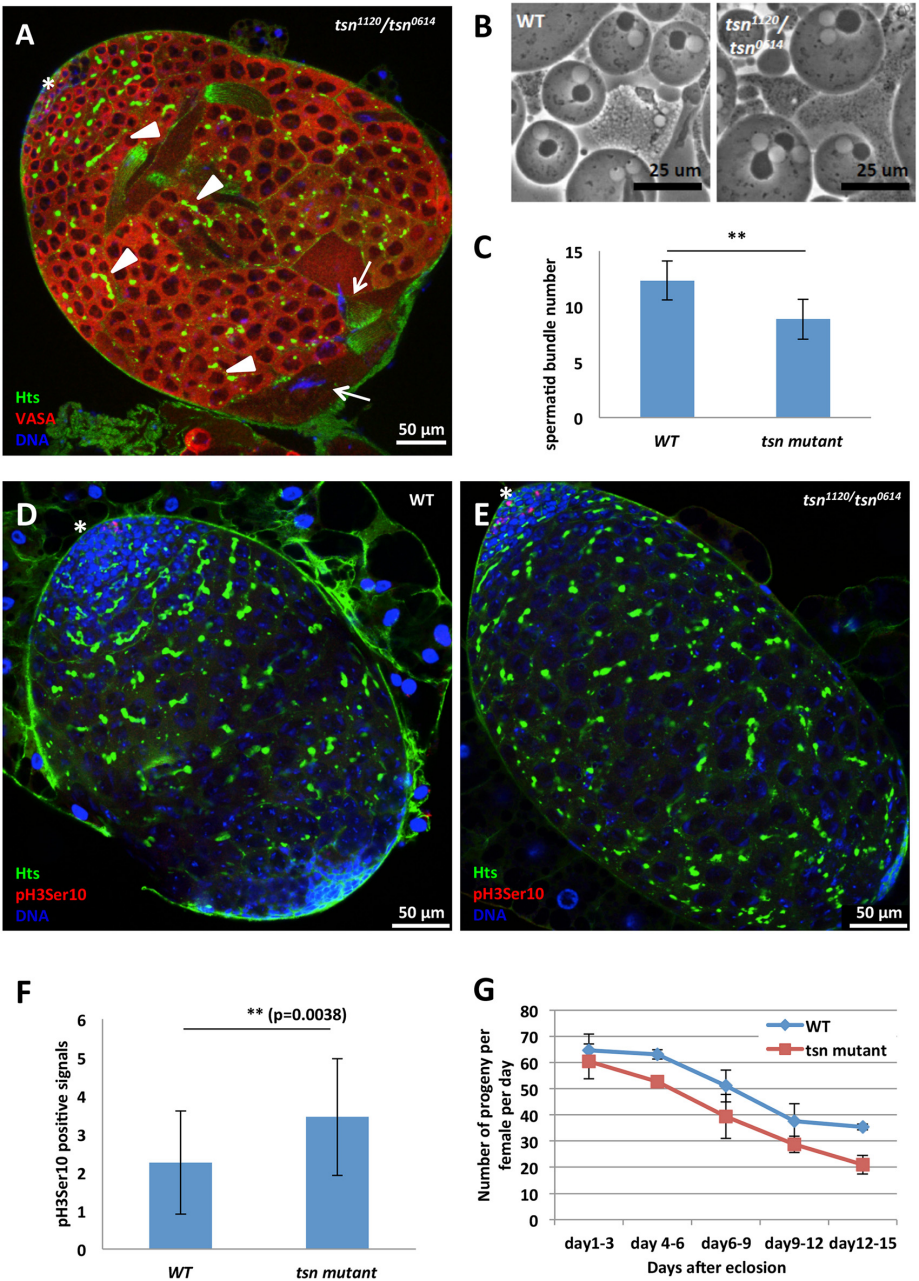
*tsn* mutations cause spermatogenic cell overproliferation, cytokinetic defects during meiosis, fewer spermatid bundles, and reduced male fertility. (**A**) The apical tip region of 2-day-old adult *tsn*^*1120*^*/tsn*^*0614*^ mutant testes. Testes were stained with anti-VASA (germ cell, red) and anti-Hts (fusome, green) antibodies. DNA was labeled by DAPI (blue). The swollen apical tip of the mutant testis was filled with VASA-positive germ cells containing branched fusomes (arrow head) and faint DNA staining, indicating these cells are spermatocytes. A few bundles of elongated spermatids with their heads (arrow) resided at the apical tip of the mutant testis, in contrast to their normal localization at the basal region of the testis. Asterisk: the hub. (**B**) Phase contrast images of round spermatids from adult WT (left) and *tsn*^*1120*^*/tsn*^*0614*^ mutant (right) testes. In mutant testes, round spermatids containing a single Nebenkern (mitochondrial derivates) associated with two haploid nuclei were observed, indicating there were meiotic cytokinesis defects (see text). (**C**) The quantification of spermatid bundle number in 2-week-old adult testes revealed that *tsn*^*1120*^*/tsn*^*0614*^ mutants had fewer spermatid bundles compared to the WT. (**D-E**) The third instar larval testes of WT and *tsn*^*1120*^*/tsn*^*0614*^ mutant were immunostained with anti-phospho-histone3 (pH3, mitotic index, red) and anti-Hts (fusome, green) antibodies. DNA was labeled by DAPI (blue). Asterisks indicate the hubs. The *tsn* mutant testis had more pH3-positive spermatogonia and was larger in size. (**F**) The quantification of pH3-positive spermatogonia per testis. The WT testes showed an average of 2.56 pH3-positive cells per testis (n = 27) and the *tsn*^*1120*^*/tsn*^*0614*^ mutant had an average of 3.44 pH3-positive cells per testis (n = 27) (see text). (**G**) The fertility of *tsn* mutant (red line) and WT (blue line) males. Initially, the *tsn*^*1120*^*/tsn*^*0614*^ mutant males showed a similar fertility as the WT controls, but the fertility was decreased faster over time compared to the WT males. Error bars represent mean ± standard error of the mean ((C) and (F) N ≧ 27; (G) N≧ 3). ** denotes P<0.01. The differences between WT and *tsn* mutant males In (G), *p* = 0.0000437 (by t-test) for the differences between WT and *tsn* mutant males.

To further investigate the spermatogenic defects of *tsn* mutants, we immunostained mutant testes from 2-day-old *tsn* ^*1120*^*/tsn* ^*0614*^ transheterozygote males with VASA as a germ-cell marker, Hu-Li Tai Shao (Hts) as a fusome marker, and DAPI as a DNA marker. Fusome is a germline-specific organelle containing membrane skeletal proteins and connecting differentiating germ cells within a germline cyst [25]. In the testis, it connects spermatocytes derived from a single gonialblast (the differentiated daughter cell of a spermatogonial stem cell). The majority of cells in the swollen apical tip were VASA positive (Fig 4A, red) with branched fusomes (Fig 4A, arrow head) and faint DNA staining signal, indicating that these excessive number of early spermatogenic cells were likely spermatocytes. In addition, we found a few bundles of elongated spermatids with their heads resided at the apical tip of the testis. 62% of the testes examined displayed this phenotype (n = 42; Fig 4A, arrow), in contrast to their normal localization at the basal region of the testis. In order to assess whether the excess spermatocytes in *tsn* mutants are resulted from the defects in GSCs, we examine the number of GSC located next to the hub, the somatic niche of GSCs in testes. The average number of GSCs per *tsn* mutant testes was 6.5 (n = 12), which was similar to that in wildtype control testes (6.67, n = 12), even though all the mutant testes displayed the swollen tip phenotype (S6 Fig). This result indicates that the spermatocyte expansion phenotype of *tsn* mutants is not caused by abnormal GSC number.

To determine whether the excess spermatocytes are due to a higher proliferation rate of spermatogonial cells, we immunostained *tsn* mutant third instar larval testes with anti-phospho-histone-3 (pH3) antibody to examine the mitotic index of spermatogonia. The mutant third instar testes show a higher mitotic index of spermatogonia and are already larger than *tsn*^+^*/tsn*^+^ wild-type larval testis, even though the mutant spermatogonia are similar in size to those in wild-type larval testes (*cf*. Fig 4D and 4E). Quantification revealed that the wild-type testes exhibited an average of 2.56 pH3-positive spermatogonia per testis (n = 27), yet the mutant testes had an average of 3.44 pH3-positive spermatogonia per testis (n = 27) (Fig 4F). The higher mitotic index of spermatogonia can be caused by two possibilities: a reduced differentiation from spermatogonia to spermatocyte and/or higher proliferation rate of spermatogonia. In the former case, the number of spermatocytes would decrease because the germ cells would be arrested at the spermatogonial stage. However, we did not observe a reduction of spermatocytes in *tsn* mutant testes. Thus, the higher mitotic index of spermatogonia indicates that there is likely a higher proliferation rate of spermatogonia in *tsn* mutant testes.

To further determine whether TSN regulates later stages of spermatogenesis, we used phase contrast microscopy to examine onion-stage spermatids in freshly squashed *tsn* mutant testes. In wild-type testes, each round spermatid contained a Nebenkern (mitochondrial derivates, Fig 4B, left panel, dark sphere) associated with a haploid nucleus (Fig 4B, left panel, white sphere). However, in 24% (n = 58) of *tsn* mutant testes, approximately 30% of round spermatids contain a single over-sized Nebenkern associated with two haploid nuclei (Fig 4B, right panel), indicating that these *tsn* mutant spermatids have succeeded in nuclear division but fail in proper cytokinesis. To assess if the spermatogenic defects are more severe in aged files, we examined the spermatid bundle numbers of 2-week-old mutant testes. The average numbers of spermatid bundle in wild-type and *tsn* mutants were 12.33 (n = 27) and 8.85 (n = 27), respectively. The reduction of spermatid bundles in the *tsn* mutants was significant (p = 2.24E-09, Fig 4C). This decreased instead of increased number of spermatid bundles in *tsn* mutant flies indicates that spermiogenesis (the process of spermatid morphogenesis) may be delayed and/or partially blocked in the mutants. Taken together, these results indicate that the *tsn* mutant testes likely have a higher proliferation of spermatogonia but a compromised differentiation of spermatocytes.

To evaluate the impact of these spermatogenic defects on fertility, we analyzed the fertility of *tsn* mutant males by crossing them to *w*^*1118*^ virgin females. Initially, the mutant males showed relatively normal fertility, but their fertility was decreased faster over time as compared to the control *tsn*^+^/*tsn*^+^ wild-type males (Fig 4G), causing a significantly reduced male fertility (P = 4.38E-05), as expected from their compromised spermatogenesis. Expression of one copy of wild-type *tsn* gene in germ cells using *nosVP16-Gal4* driver in *tsn* mutant males rescued their fertility to 74% of wildtype males at 2wk-old age, as effective as introducing one copy of the endogenous wildtype *tsn* gene (on the *TM3* balancer) into the *tsn* mutant background (S5B Fig). These results indicate the germline-dependence of *tsn* function in the testis. In addition, these results indicate the dose-dependence of the TSN function in spermatogenesis.

To further study the dose-dependence of TSN in spermatogenesis, we overexpressed TSN in either germline cells or all cells in wildtype testes and examined the potential phenotypical consequence (S8 and S9 Figs). No obvious abnormality was observed, except that only a slight increase of hub cells number was observed in *Actin*-*gal4*>*Flag-tsn* testes (S8 and S9B Figs). Hence, increasing TSN level to more than wildtype level does not generate more impact on germ cell differentiation.

### Piwi and TSN antagonistically interact in the germline to regulate spermatogenesis

**Fig 5:**
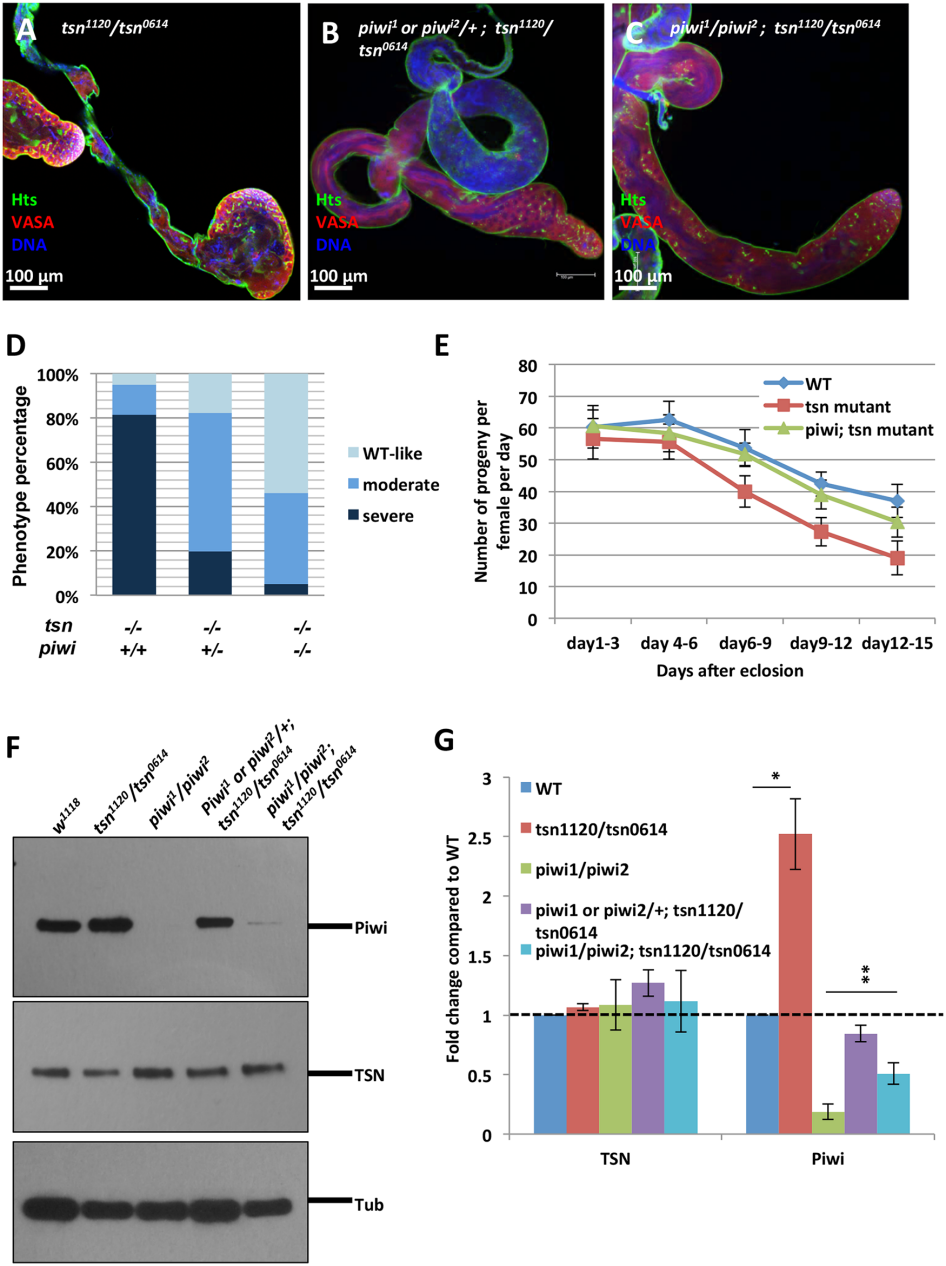
TSN antagonizes Piwi during spermatogenesis. (**A-C**) Immunostaining of Hts (green) and VASA (red) in testes from *tsn* mutants, and *piwi* and *tsn* double mutants. The defects of *tsn* mutant testes were rescued by *piwi* mutations in a dosage-dependent manner. (**D**) Quantification of the results in A-C. The phenotype is categorized into three classes: WT-like (as shown in C), moderate (as shown in B), and severe (as shown in A) phenotype. In *tsn*^*1120*^*/tsn*^*0614*^ mutant testes, only 5% of the testes displayed WT-like phenotype, 14% of the testes revealed moderate phenotype, and 81% of the testes showed severe phenotype. However, introducing one copy of *piwi*^*1*^ resulted in 18% and 63% of the testes showing WT-like and moderate phenotype, respectively. Introducing *piwi*^*1*^*/piwi*^*2*^ led to 54% and 41% of the testes showing WT-like and moderate phenotype, respectively. (N≧50 for each of the genotypes) (**E**) Fertility assay of *piwi* and *tsn* double mutant males. The reduced fertility of *tsn* mutant males (red line) was restored by introducing *piwi* mutations to the *tsn* mutant background (green line). The fertility of WT males is shown as the blue line. (**F**) Western blot analysis of Piwi expression level in *piwi* mutant, *tsn* mutant, and *piwi* and *tsn* double mutant testes. Piwi protein level was upregulated in *tsn* mutant testes, suggesting a negative regulation of TSN on Piwi expression. (G) qRT-PCR of Piwi and TSN in *piwi* mutant, *tsn* mutant, and *piwi* and *tsn* double mutant testes. Piwi mRNA level was upregulated in *tsn* mutant testes, indicating TSN negatively regulates Piwi expression at the mRNA level. Error bars represent mean ± standard error of the mean (N ≧ 3). **P<0.01.

To investigate the biological significance of TSN and Piwi interaction in regulating spermatogenesis, we examined the phenotype of the *piwi* and *tsn* double mutants. We focused on *piwi*^*1*^ and *piwi*^*2*^ mutant alleles, which are strong recessive mutations of *piwi* [5, 6]. Remarkably, we found that introducing one or two copies of either *piwi*^*1*^ or *piwi*^*2*^ mutant alleles into the *tsn* mutant background rescued *tsn* mutant phenotype in a dosage-dependent manner (Fig 5A–5C). We categorized the phenotype into three classes based on the extent of swollen apical tip of the testis: severe (as shown in Fig 5A), moderate (as shown in Fig 5B), and WT-like (as shown in Fig 5C) phenotype. In *tsn*^*1120*^*/tsn*^*0614*^ mutant testes, only 5% of the testes showed WT-like morphology, 14% of the testes had moderate phenotype, and 81% of the testes displayed severe phenotype. However, introducing one copy of *piwi*^*1*^ allele resulted in 18% and 63% of the testes showing WT-like and moderate phenotype, respectively. Introducing *piwi*^*1*^*/piwi*^*2*^ alleles led to 54% and 41% testes showing WT-like and moderate phenotype, respectively (Fig 5D). Moreover, the fertility of *tsn* mutants (Fig 5E, red line) was also restored by *piwi* mutations (Fig 5E, green line). Interestingly, Western blot and quantitative RT-PCR (qRT-PCR) analyses revealed that the protein and mRNA expression levels of *piwi* were upregulated in *tsn* mutant testes (Fig 5F and 5G). Immunostaining of Piwi in *tsn* mutant testes further showed that the expression pattern of Piwi did not change compared to that in the control (S7 Fig). Therefore, the upregulation of Piwi is not due to ectopic expression of Piwi, but is mostly, if not completely, due to increased Piwi expression within its expressing cells. Consistently, overexpression of TSN with *actin5c-Gal4* driver in testes leads to slight decrease in Piwi expression, with no effect in Piwi localization (S9 Fig). The antagonistic interactions between Piwi and TSN at both genetic and molecular levels together define a functionally antagonistic relationship between TSN and Piwi in spermatogenesis.

Interestingly, we observed similar regulatory relationship of Piwi and TSN in the ovary. Although *tsn* mutant ovaries did not show any obvious oogenesis defects (S4C Fig), the germline-depletion phenotype of *piwi* mutants (S4B Fig) was partially restored by introducing the mutations of *tsn* (S4D Fig). Furthermore, Piwi protein and mRNA expression levels were both significantly upregulated in the *tsn* mutant ovaries (S10 Fig), suggesting the antagonistic interaction between Piwi and TSN is conserved in spermatogenesis and oogenesis. Because TSN is involved in RNA metabolism [14–18], we performed TSN RNA immuneprecipitation using Flag antibody to examine if TSN binds to Piwi mRNA (see Materials and Methods). Piwi mRNAs are enriched more than 7-fold using two different sets of *piwi* primers (S10C Fig). These results together indicate that TSN regulate Piwi expression likely by directly binding to its mRNA.

**Fig 6:**
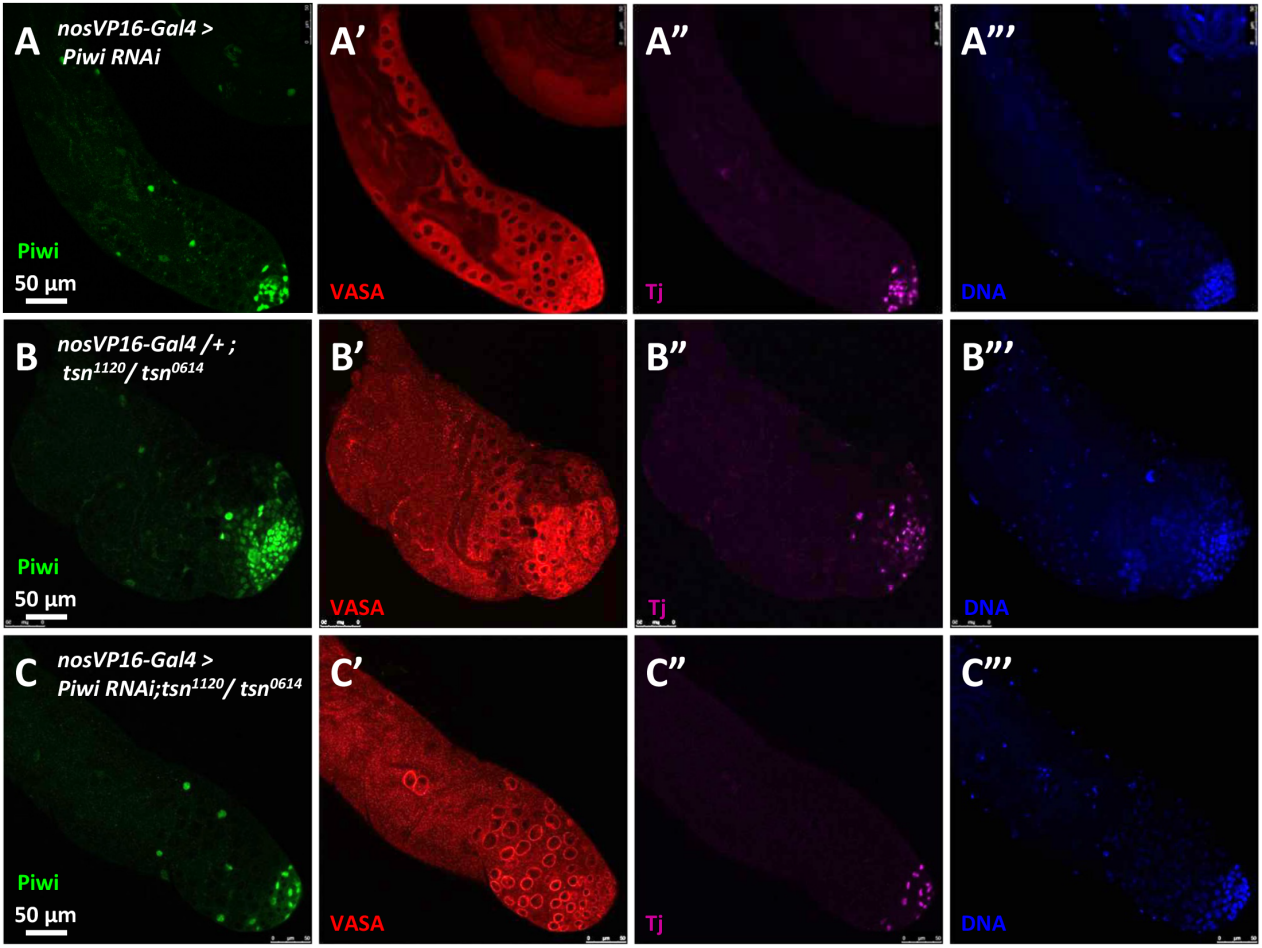
TSN antagonizes the germline function of Piwi. The testes were stained with anti-Piwi (green), anti-VASA (germ cell, red), and anti-Tj (somatic cells, purple) antibodies. DNA was labeled by DAPI (blue). (**A-A‴**) Germline knockdown of Piwi using *nosVP16-Gal4* driver. (**B-B‴**) The *tsn*^*1120*^*/tsn*^*0614*^ mutant displayed a swollen apical tip of the testis. (**C-C‴**) The depletion of Piwi in germ cells rescued *tsn* mutant phenotype, suggesting TSN antagonizes the germline function of Piwi in regulating spermatogenesis.

As Piwi is expressed in both germline and somatic cells, we next asked whether TSN antagonizes the germline or the somatic function of Piwi. To address this question, we used *nosVP16-Gal4* (germline) and *tj-Gal4* (somatic) drivers to knockdown Piwi tissue-specifically in the *tsn* mutant background. Germline knockdown of Piwi rescued *tsn* mutant phenotype (72% of the testes examined were WT-like, n = 75; Fig 6); however, somatic depletion of Piwi did not (S11 Fig). These results suggest that TSN antagonizes the function of Piwi in germ cells to regulate spermatogenesis.

### TSN synergistically interacts with Piwi to silence transposons but has little role in piRNA biogenesis

**Fig 7:**
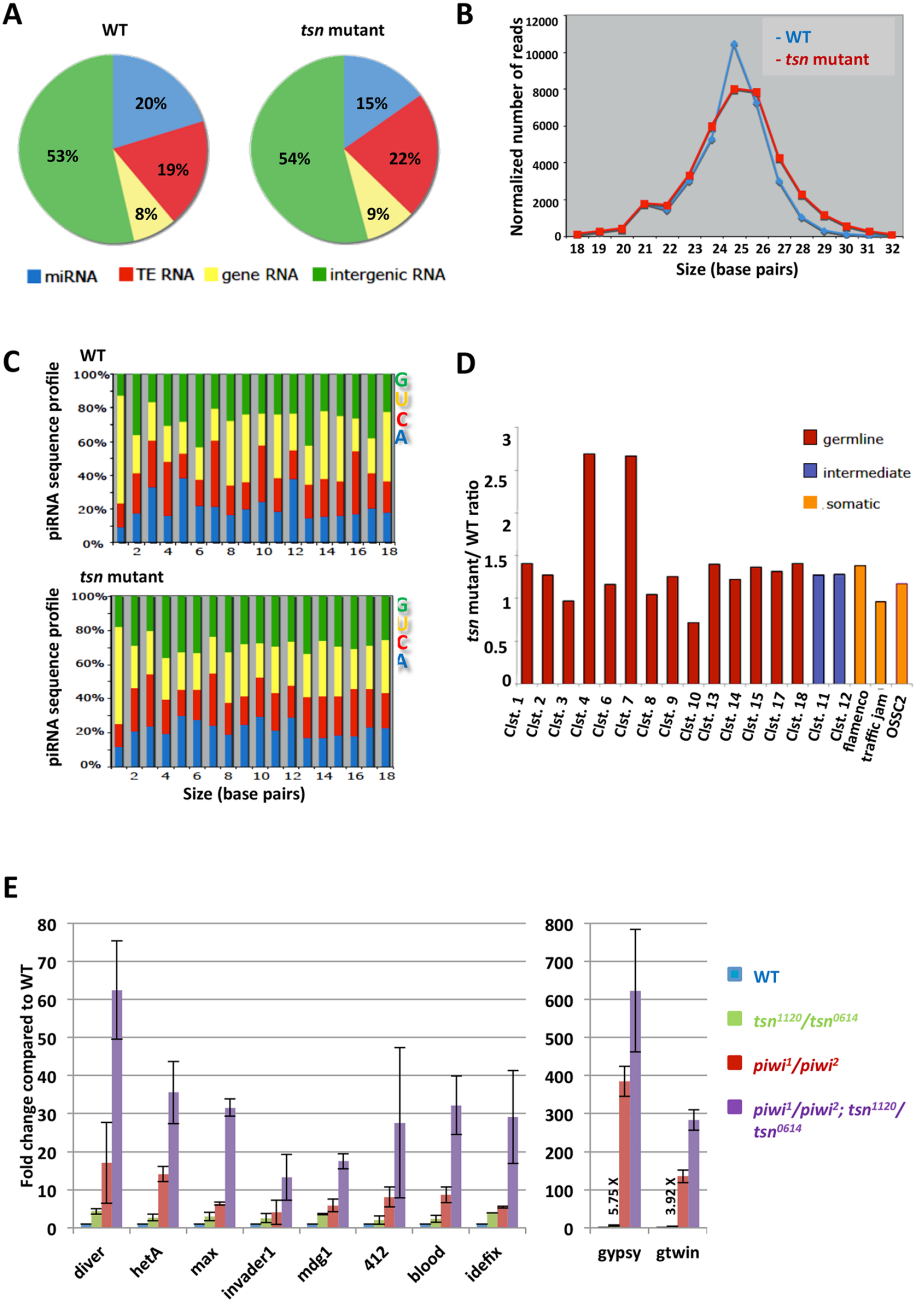
TSN shows only subtle effects on piRNAs and transposon silencing. (**A**) Small RNA composition in the WT (left) and *tsn*^*1120*^*/tsn*^*0614*^ mutant (right) testes. TE RNA, small RNAs derived from transposon regions; gene RNA, small RNAs derived from gene-coding regions; intergenic RNA, small RNAs derived from intergenic regions. (**B**) Size distribution of small RNAs (excluding miRNAs and fragments of long cellular RNAs such as tRNAs and rRNAs) in the WT (blue) and *tsn*^*1120*^*/tsn*^*0614*^ mutant (red) testes. The level of 25-nt small RNAs was slightly decreased in the *tsn* mutants. (**C**) Nucleotide compositions of small RNAs (excluding miRNAs and fragments of long cellular RNAs such as tRNAs and rRNAs) in the WT (top) and *tsn*^*1120*^*/tsn*^*0614*^mutant (bottom) testes. The 1U-bias piRNA signature and nucleotide composition were not affected in *tsn* mutants. (**D**) Relative abundance of small RNAs derived from 19 previously reported piRNA clusters in *tsn* mutant testes compared to the WT testes are shown. Small RNAs uniquely mapped to these clusters were used for this analysis. These clusters are categorized into three groups according to the expression preference of their piRNAs [39–41], with the germline-enriched class colored in red, the intermediate class colored in blue, and the soma-enriched class colored in orange. No strong impact of *tsn* mutations on these piRNA clusters was observed (see text). (**E**) qRT-PCR was performed to determine the expression of multiple transposons, relative to *rp49*, in *tsn*^*1120*^*/tsn*^*0614*^ mutant testes. Transcript levels from the WT testes were set as 1, and fold-changes are indicated. Error bars represent mean ± standard error of the mean (N = 3). *tsn* mutants showed very moderate defects in transposon silencing (see text).

Because Piwi plays an important role in the piRNA pathway [2], we wanted to know whether TSN regulates piRNA biogenesis and functions. To address this question, we first sequenced small RNA libraries prepared from an equal quantity of two-day-old *tsn*^+^*/tsn*^+^ wild-type and *tsn*^*1120*^*/tsn*^*0614*^ mutant testes. The composition of small RNA libraries from wild-type and *tsn* mutant testes are shown in Fig 7A. The size distribution profiles of small RNAs (excluding miRNAs and fragments of long cellular RNAs such as tRNAs and rRNAs) from wild-type and *tsn* mutant testes are shown in Fig 7B. It revealed that the *tsn* mutant testes have essentially identical piRNA profiles (Fig 7B). Moreover, the 1U-bias of piRNAs (a strong U bias at the most 5’ position) and the nucleotide composition were well maintained in the *tsn* mutants (Fig 7C). We further mapped all of the small RNAs to known piRNA clusters, and only clusters 4 and 7 showed ~3-fold upregulation in *tsn* mutants (Fig 7D). However, we noticed that relatively few deep sequence reads mapped to these two clusters, so the 3-fold increase could be due to or contributed by fluctuation in reads. These data indicate that, unlike Piwi, TSN either is not involved in piRNA biogenesis or plays only a very minor role in this process.

To investigate whether TSN is involved in piRNA biogenesis in the ovary and to test whether the mild effect of *tsn* mutations on small RNAs in the testis is caused by altered abundance of different cell types in the mutant testis, we sequenced and analyzed small RNAs prepared from wild-type and *tsn* mutant ovaries. The total small RNA composition profiles were shown in S12A Fig. Similar to the results observed in testes, the size distribution profiles of small RNAs from wild-type and *tsn* mutant ovaries are not significantly affected (S12B Fig). The 1U-bias and nucleotide composition were not affected in the mutant ovaries, either (S12C Fig). We next examined the abundance of piRNAs generated from known piRNA clusters, and did not observe any strong effects of *tsn* mutations on these piRNA clusters (S12D Fig). Together, these results indicate that TSN has a minor role, if any, in piRNA biogenesis in both testes and ovaries.

To exclude the possibility that the compensation of other Piwi-interacting Tudor proteins may rescue the loss of TSN, we examine the expression level of Papi and Tudor [22] in *tsn* mutant testes, and did not detect significant difference in their expression (S13 Fig). Therefore, TSN unlikely to be directly involved in piRNA biogenesis.

Because a primary function of Piwi is to silence transposons [2], we next examined whether *tsn* mutations results in any transposon desilencing and whether TSN interacts with Piwi in regulating transposons. To this end, we analyzed the expression of transposons in wild-type, *tsn* mutant, *piwi* mutant, and *piwi* and *tsn* double mutant testes by qRT-PCR. Consistent with the only slight reduction of piRNAs in *tsn* mutant testes shown in Fig 7B, transposons were only mildly upregulated (~2-6X change) in the *tsn* mutants compared with the wild-type controls (Fig 7E). In contrast, the upregulation of transposons in the *piwi* mutant is more significant (~4-18X for most transposons and 120X and 385X for *gtwin* and *gypsy*, respectively; Fig 7E). Remarkably, in *tsn; piwi* double mutants, the transposon upregulation is even much more drastic (~13-62X for most transposons and 280X and 620X for *gtwin* and *gypsy*, respectively). Similar results were observed in ovaries (S12E Fig). These results indicate that TSN and Piwi interact synergistically in transposon silencing in both testes and ovaries.

## Discussion

Piwi is a multifunctional protein and its deficiency leads to abolishment of GSC self-renewal, abnormalities in germ cell proliferation and differentiation, defects in piRNA biogenesis, derepression of transposons, and aberrant epigenetic programming (reviewed in [1, 2]). However, it has not been reported that these functions are separable. In addition, no negative interactor of Piwi proteins has been identified. Here we have identified TSN as a novel Piwi interactor that interacts with Piwi antagonistically and demonstrated that this interaction is only for spermatogenesis but not for piRNA biogenesis or transposon silencing. The *tsn* mutant testis exhibits phenotypic defects, including germline overproliferation, meiotic cytokinesis abnormality, and a reduced number of spermatids, all of which contribute to a decrease in the fertility of these mutant males (Fig 4). The opposite phenotype of *piwi* and *tsn* mutants in germ cell proliferation, together with the negative regulation of Piwi by TSN, indicate an antagonistic relationship between Piwi and TSN that is essential for spermatogenesis. In support of this, the phenotype of *tsn* mutants in spermatogenesis and fertility is rescued by *piwi* mutations (Fig 5A–5E). Because TSN negatively regulates the expression of *piwi* mRNA and Piwi protein in the testis (Fig 5F and 5G), it is likely that the antagonistic interaction happens at level of Piwi expression. Furthermore, this regulation occurs mostly, if not exclusively, in the germline, since TSN function is germline-dependent (S5B Fig) and *tsn* phenotype is rescued by germline-specific *piwi* knockdown (Fig 6).

It is worth noting that the TSN function in spermatogenesis is not only germline dependent but also dose-dependent. *Tsn*/+ testes show intermediate degrees of defects between *tsn*/*tsn* and +/+ testes. Furthermore, the *nos-Gal4*-driven expression of one copy of *tsn* gene rescues defects of *tsn*/*tsn* mutants to that of *tsn*/+ testes. However, over-expression of TSN in either the germline or the entire testis does not have detectable effect, which suggests that the dose-dependence is capped at the +/+ level.

Our study indicates that the antagonistic regulatory relationship between Piwi and TSN is conserved between spermatogenesis and oogenesis. TSN also negatively regulates the expression of *piwi* mRNA and Piwi protein in the ovary (S10 Fig). Furthermore, TSN binds to the *piwi* mRNA in the ovary (S10C Fig). These results also support that TSN antagonizes Piwi function at least in part by directly binds to its mRNA and suppress its expression.

Despite the strong interaction between TSN and Piwi, it is surprising that TSN does not have obvious effects on piRNA biogenesis (Fig 7). Although a simple explanation would be that spermatogenesis is more sensitive to the dose of Piwi than piRNA biogenesis. This is unlikely because Piwi expression is up-regulated in *tsn* mutant, yet *Drosophila* strains with four copies and even six copies of *piwi* are perfectly fertile and can be maintained as stocks [7]. Therefore, we favor an alternative possibility that the antagonistic protein-protein interaction between TSN and Piwi is needed for spermatogenesis but not for piRNA biogenesis,

It is surprising to observe that, despite the antagonistic interaction between TSN and Piwi, they have synergistic effect on transposon silencing. This might reflect that TSN and Piwi act through independent pathways that silence transposons (S14 Fig). In *piwi* mutants, transposons are significantly de-silenced, as previously reported by many. Interestingly, *tsn* mutants display a milder de-silencing effect. This is likely because Piwi is significantly up-regulated in *tsn* mutants, which partially compensated for the loss of TSN-mediated transposon repression. In *tsn; piwi* double mutants, both TSN and Piwi-mediated repression mechanisms are abolished, which leads to an even more drastic transposon repression that has not been reported. Thus, PIWI/piRNA-mediated mechanism is not the only mechanism involved in transposon repression. It would be important to investigate the TSN-mediated transposon silencing mechanism and to determine whether it depends on piRNA.

Relevantly, the Piwi-TSN protein interaction may only affect piRNA-independent function of Piwi such as miRNA regulation [7] or other unidentified roles of Piwi. Both TSN and Piwi have been implicated in the miRNA pathway [7, 26]. Since miRNAs play an important role during germline development, it would be intriguing to examine whether TSN interacts with Piwi to mediate miRNA regulation which in turn exerts different effects on spermatogenesis and transposon silencing. In any case, the above data allow us to conclude that the function of Piwi in spermatogenesis can be separated from piRNA biogenesis and transposon silencing.

It has been shown that the expression of human TSN (a.k.a., SND1) is upregulated in various human cancers such as colon, prostate, breast, and hepatocellular cancers [27–30]. In prostate cells, SND1 recruits splicing factor SAM68 and other spliceosomal components on CD44 pre-mRNA, promotes the inclusion of CD44 variable exons, which correlates with increased proliferation, motility, and invasiveness of cancer cells [27]. Moreover, SND1 interacts with Metadherin (MTDH) to promote metastasis and was also shown to promote resistance to apoptosis and to regulate the expression of genes associated with metastasis and chemoresistance [28]. However, both the function of TSN during normal development and the molecular mechanism of TSN in cancer cells still remain elusive. Importantly, PIWI proteins are also implicated in cancers [31–33]. Therefore, our study of TSN and Piwi interaction provides a possible mechanism in tumorigenesis, and it may be due to the multifunctional nature and the broad interacting partners and targets of TSN.

## Materials and Methods

### *Drosophila* stocks, transgene expression, and RNAi

All fly stocks were raised at 25°C on yeast-containing molasses/agar medium. Both *yw* and *w*^*1118*^ flies were used as the wild-type controls. The *tsn* EMS mutant alleles were generated and screened using Fly TILLING [24]. The deficiency allele that disrupts the *tsn* genomic region, *w*^*1118*^; *Df(3L)BSC125/TM6B*, *Tb*^*1*^, was obtained from Bloomington Stock Center. The *tsn* RNAi strain HMS00184 was obtained from TRiP at Harvard. The knockdown of *tsn* was induced by *Actin5C-Gal4* driver obtained from Bloomington *Drosophila* stock center. UASp-driven Flag-tagged *tsn* transgenic strains, carrying a functional *tsn* gene, were generated with standard protocols using pVALIUM10-roe vector from TRiP at Harvard. Germline-specific expression of the Flag-tagged *tsn* transgene was achieved by introducing *nosVP16-Gal4* (Bloomington *Drosophila* stock center) and Flag-tagged *tsn* transgene in the *tsn*^*1120*^*/tsn*^*0614*^ mutant background. *piwi*^*1*^ and *piwi*^*2*^ alleles were generated by P-element insertions as previously described [5, 6]. The *piwi* RNAi strain *w*^*1118*^; *P{GD11827}v22235* was obtained from Vienna *Drosophila* RNAi Center. The knockdown of *piwi* was induced by *nosVP16-Gal4* driver (Bloomington *Drosophila* stock center) and *tj-Gal4* driver (*Drosophila* Genetic Resource Center).

### Biochemical purification and identification of cytoplasmic Piwi interacting peptides

All chromatographic steps and mass spectrometry analysis were described previously [23].

### Embryo extraction and Immunoprecipitation

Cytoplasmic extracts of *w*^*1118*^ embryos were collected as described previously [23]. All steps in extract preparation were performed at 4°C. A total of 1 mg of total protein in a volume of 1 ml H(0.15) buffer (25 mM HEPES-NaOH, pH 7.8, 150mM NaCl, 0.5 mM EGTA, 0.1 mM EDTA, 2 mM MgCl2, 0.02% NP-40 and 20% glycerol) was used for each immunoprecipitation reaction. The lysates were pre-cleared using Protein A/G PLUS-Agarose (Santa Cruz) for 1h at 4°C. Pre-cleared lysates were incubated with the monoclonal mouse anti-Piwi antibody (a gift from M.C. Siomi)[34] or the monoclonal mouse anti-TSN antibody, a gift from O. Silvennoinen [35], overnight at 4°C with gentle agitation. 60 μl of beads were then added to the lysate-antibody mixture and incubated further for 3 h at 4°C. Beads were then washed five times with 1 ml of H(0.15) buffer at 4°C and finally analyzed by Western blotting.

### Western blot analysis

For Western blot analysis, the following antibodies were used: monoclonal mouse anti-Piwi (1:200), a gift from M.C. Siomi [34], monoclonal mouse anti-TSN (1:5000), a gift from O. Silvennoinen [35], polyclonal rabbit anti-GAPDH antibody (1:5000, Sigma), polyclonal Guinea Pig anti-Papi antibody (1:5000) and polyclonal mouse anti-β-tubulin (1:10000, Developmental Studies Hybridoma Bank) antibodies. Peptides corresponding to the 26 residues of the N-terminal and the 27 residues of the C-terminal of *Drosophila* TSN were synthesized and used for generating polyclonal rabbit anti-TSN-N and anti-TSN-C antibodies, respectively (Cocalico Biologicals). Both antibodies were purified and used at 1:5000 dilution. The specificity of each anti-TSN antibody is shown in S1 Fig.

### Immunostaining

The preparation of embryos, ovaries, and testes was performed as previously described [25, 36]. For immunofluorescence staining, the following primary antibodies were used: monoclonal mouse anti—Flag M2 (1:500; Sigma-Aldrich), monoclonal mouse anti-Piwi (1:20), a gift from M.C. Siomi [34], monoclonal mouse anti-TSN (1:500), a gift from O. Silvennoinen [35], polyclonal rabbit anti-VASA (1:400, Santa Cruz), polyclonal rabbit anti-Ser10-phospho-histone H3 (1:200, Cell Signaling), polyclonal rabbit anti-TSN-N and anti-TSN-C (both at 1:1000 dilution), polyclonal mouse anti-Hts (1:40, Developmental Studies Hybridoma Bank), and polyclonal guinea pig anti-Tj (1:100, a gift from D. Godt, University of Toronto) antibodies. Alexa Fluor -488, -568, or -633-conjugated goat anti-rabbit, anti-guinea pig, and anti-mouse IgG secondary antibodies were purchased from Jackson Immunoresearch Laboratory and were used at 1:400 dilution. Immunofluorescently labeled samples were counterstained with DAPI with standard protocol. Images were taken using Leica TCS SP5 Spectral Confocal Microscope in the sequential scanning mode and then processed using Photoshop (Adobe).

### Live testicular squashes and phase contrast microscopy

Testes were dissected from 3-day-old adults in phosphate-buffered saline (PBS) and transferred into a 2-μL drop of PBS on a coverslip. The testes were then torn open using tungsten needles and slightly squashed by putting a slide over the coverslip. Phase contrast images of squashed testes were obtained using a Leica DM6000 microscope with a 40X objective.

### Male fertility assay

Individual 2-day-old males of the indicated genotypes were crossed with three 2-day-old virgin *w*^*1118*^ females. For every 3 days, each vial was visually examined to ensure eggs had been laid. The females were then discarded and each male was transferred to a new vial and mated with three 2-day-old virgin females. The number of adult progeny in each vial was counted on the 20^th^ day after each mating. A minimum of 15 males for each genotype was used for testing.

### Small RNA cloning and analysis

Low-molecular-weight RNAs were isolated from the adult testes of *w*^*1118*^ and *tsn*^*1120*^*/tsn*^*0614*^ mutant males (approximately 200 pairs for each genotype) using a mirVana miRNA isolation kit (Life Technologies). Small RNAs ranging in size between 16 and 29 nucleotides (nt) (below 2S rRNA) were gel-purified, and small RNA libraries were prepared using a small RNA sample prep kit (Illumina) according to the alternative v1.5 protocol. The clones were sequenced using Genome Analyzer II. Only sequences perfectly matching the *Drosophila melanogaster* release 5 genome (excluding Uextra) were analyzed. As TSN is known to regulate miRNAs [17], here we used the total reads instead of the reads of miRNAs to normalize the two libraries. After removal of miRNAs and fragments of long cellular RNAs such as tRNAs and rRNAs, we mapped 18–32-nt unique piRNAs to a subset of known piRNA clusters and to the complete collection of *Drosophila melanogaster* transposable elements (Repbase) [37].

### Total RNA extraction and quantitative RT-PCR

Thirty pairs of testes were dissected from 2-day-old adults in ice-cold PBS. Total RNA was then extracted using TRIzol (Invitrogen) following manufacturers’ protocols. RNA was finally eluted in 30μl nuclease-free water and quantified by Nanodrop. 2 μg of eluted RNA was used to generate cDNA in a 20 μl reaction using High Capacity cDNA Reverse Transcription kit (Applied Biosystems).

Quantitative RT-PCR of the Piwi, TSN, and transposon transcripts was performed as previously described [38]. The following primer pairs were used- Piwi: forward (5’-TGCCATGAGCAGTTATACGC-3’) and reverse (5’-TGTCCA GTTCCATGTTCCAG-3’); TSN: forward (5’-GGCAACATGTGTTCGCTATC-3’) and reverse (5’- GTGGCGTTATCCTTCTTTGC-3’); 412: forward (5’-CACCGGTTTGGTCGAAAG-3’) and reverse (5’-GGACATGCCTGGTATTTTGG-3’); blood: forward (5’-TGCCACAGTACCTGATTTCG-3’) and reverse (5’-GATTCGCCTTTTACGTTTGC-3’); diver: forward (5’-GGCACCACATAGACACATCG-3’) and reverse (5’-GTGGTTTGCATAGCCAGGAT-3’); gtwin: forward (5’-TTCGCACAAGCGATGATAAG-3’) and reverse (5’-GATTGTTGTACGGCGACCTT-3’); gypsy: forward (5’-GTTCATACCCTTGGTAGTAGC-3’) and reverse (5’-CAACTTACGCATATGTGAGT-3’); HetA: forward (5’-CGCGCGGAACCCATCTTCAGA-3’) and reverse (5’-CGCCGCAGTCGTTTGGTGAGT-3’); invader1: forward (5’-GTACCGTTTTTGAGCCCGTA-3’) and reverse (5’-AACTACGTTGCCCATTCTGG-3’); max: forward (5’-TCTAGCCAGTCGAGGCGTAT-3’) and reverse (5’-TGGAAGAGTGTCGCTTTGTG-3’); mdg1: forward (5’-AACAGAAACGCCAGCAACAGC-3’) and reverse (5’-CGTTCCCATGTCCGTTGTGAT-3’); rt1a: forward (5’-CCACACAGACTGAGGCAGAA-3’) and reverse (5’-ACGCATAACTTTCCGGTTTG-3’); rp49: forward (5’-CCGCTTCAAGGGACAGTATCTG-3’) and reverse (5’-ATCTCGCCGCAGTAAACGC-3’).

### Immunoprecipitation and quantitative RT-PCR of TSN-associated RNA

Adult testes from *wildtype* or *actin5c-Gal4>UASp Flag-TSN* were dissected in ice-cold PBS and homogenized in H(0.15) buffer. Testes lysates were collected by centrifugation at 10,000g for 30 min at 4°C. Immunoprecipitation was performed using mouse anti-Flag (Sigma) with Protein G magnetic beads (Invitrogen). Immunoprecipitates were washed five times with H(0.15) buffer at 4°C. Immunoprecipitated RNAs were then isolated from the immunopurified complexes with Trizol and precipitated with ethanol. Reverse transcription and qRT-PCR were then done following the protocol described above. Primer pairs used are the same as described above except piwi #2: forward (5’- TGCCATGAGCAGTTATACGC-3’) and reverse (5’- TGTCCAGTTCCATGTTCCAG-3’).

## Supporting Information

S1 Fig Specificity of anti-TSN antibodies. Western blot analysis using the ovarian lysates prepared from *tsn* knockdown and control females. The blots were probed with polyclonal rabbit anti-TSN-N (left), polyclonal rabbit anti-TSN-C (middle), and monoclonal mouse anti-TSN (right) antibodies. TSN is ~100 kDa.
(TIF)

S2 Fig The knockdown of *tsn* resulted in the swollen apical tip of the testis. **(A)** Western blot analysis using mouse anti-TSN antibody showing the knockdown efficiency of *tsn* in testes. **(B)** Testes from *tsn* knockdown and control males were immunostained with mouse anti-TSN antibody. DNA was labeled by DAPI (blue). The knockdown of *tsn* caused the swollen apical tip of the testis (see text). Arrow indicates a bundle of elongated spermatids with their heads resided at the apical tip region of the testis.
(TIF)

S3 Fig The knockdown of *tsn* did not cause any obvious phenotype in ovaries. **(A)** Western blot analysis using mouse anti-TSN antibody showing the knockdown efficiency of *tsn* in ovaries. **(B)** Ovaries from *tsn* knockdown and control females were immunostained with mouse anti-TSN antibody. DNA was labeled by DAPI (blue). The knockdown of *tsn* did not generate any obvious defects in oogenesis.
(TIF)

S4 Fig TSN antagonizes Piwi during oogenesis. Immunostaining of VASA (green) and Hts (red) and in ovaries from WT (A-A‴), *piwi* mutants (B-B‴), *tsn* mutants (C-C‴), and *piwi* and *tsn* double mutants (D-D‴). The defect of *piwi* mutant ovaries (B-B‴) was significantly rescued by *tsn* mutations (D-D‴). This antagonistic relationship between TSN and PIWI in oogenesis parallels that in spermatogensis (Fig 4A–4D).
(TIF)

S5 Fig Germline expression of *tsn* transgene rescued *tsn* mutant phenotype and fertility. (A). Flag-TSN was expressed in germ cells by *nosVP16-Gal4* in the *tsn*^*1120*^*/tsn*^*0614*^ mutant background. The testes were immunostained with anti-Flag (Flag-TSN), anti-Tj (somatic cells), and anti-VASA (germ cells) antibodies. DNA was labeled by DAPI (blue). Asterisk indicates the hub. *tsn* mutant phenotype was rescued by the expression of full-length WT TSN. (B). Fertility assay of *piwi* and *tsn* double mutant males. The reduced fertility of *tsn* mutant males (red line) was restored largely by expression of Flag-tagged TSN in the germline cells driven by VP16-NosGal4 driver at the *tsn* mutant background (gray line). The fertility of WT males is shown as the light blue line.
(TIF)

S6 Fig GSC number is normal in adult *tsn* mutant testes. Germ cells were labeled with anti-VASA antibody (red) and DNA was labeled by DAPI (blue). The average numbers of GSCs in WT and *tsn* mutant testes was 6.67 (n = 12) and 6.5 (n = 12), respectively. There were no obvious abnormalities in GSCs of *tsn* mutant testes, suggesting the phenotype of *tsn* mutants may not be due to defects in GSCs.
(TIF)

S7 Fig The upregulation of Piwi caused by the mutations of *tsn* is within the Piwi-expressing cells. Immunostaining of Piwi in testes from adult WT and *tsn* mutant males. DNA was labeled by DAPI. Piwi was expressed in early germ cells and somatic cyst cells in both WT and *tsn* mutant testes. This result suggests the upregulation of Piwi is in the Piwi-expressing cells, but not caused by ectopic expression of Piwi.
(TIF)

S8 Fig Overexpression of *tsn* in germ cells of the wildtype testis. Germline-specific expression of the Flag-tagged *tsn* transgene was induced by *nosVP16-Gal4* in *w*^*1118*^ male flies. (A, B) Testes from 2-day-old males were immunostained with mouse anti-Flag M2 antibody. (A’, B’) DNA was labeled by DAPI (blue). (A”, B”) merged images for Flag and DAPI staining. Asterisks indicate the hubs. Overexpression of *tsn* in WT germ cells does not cause any obvious phenotype.
(TIF)

S9 Fig Overexpression of *tsn* in both germline and somatic cells of the wild-type testis. Overexpression of Flag-tagged *TSN* was induced by *Actin5C-Gal4* in *w*^*1118*^ male flies. 2-day-old testes were used for the analysis. (A) Western analysis of TSN and Piwi. Overexpressed Flag-TSN was examined with mouse anti-Flag M2 antibody. Piwi expression level is slightly reduced in TSN overexpressed testes comparing to WT testes. (B) Immunostaining of Flag-TSN and Piwi in Flag-TSN overexpressing testes. DNA was labeled by DAPI (blue). Asterisks indicate the hubs. Piwi localization is normal while its expression level especially in somatic cyst cells is reduced.
(TIF)

S10 Fig TSN negatively regulates Piwi expression at the mRNA level in ovaries. (**A**) Western blot analysis of Piwi expression level in *piwi* mutant, *tsn* mutant, and *piwi* and *tsn* double mutant ovaries. Piwi protein level was upregulated in *tsn* mutants, suggesting a negative regulation of TSN on Piwi expression. (B) qRT-PCR of Piwi and TSN in *piwi* mutant, *tsn* mutant, and *piwi* and *tsn* double mutant ovaries. Piwi mRNA level was upregulated in *tsn* mutant ovaries, indicating TSN negatively regulates Piwi expression at the mRNA level. (C). TSN binds to *piwi* mRNA in *Drosophila* ovaries. qRT-PCR to detect *piwi* mRNA (using two sets of primers: *piwi* and *piwi#2*) *and tsn* mRNA from RNA co-immunoprecipitated from wildtype and Flag-TSN overexpressing ovaries using anti-Flag antibody. Error bars represent mean ± standard error of the mean (N ≧ 3). *P < 0.05 and **P<0.01.
(TIF)

S11 Fig Somatic knockdown of Piwi does not rescue *tsn* mutant phenotypes. The testes were immunostained with anti-Piwi (green), anti-VASA (germ cell, red), and anti-Tj (somatic cells, purple) antibodies. DNA was labeled by DAPI (blue). **(A-A‴)** Somatic Piwi was knockdown using *tj-Gal4* driver. The knockdown of somatic Piwi resulted in an expansion of Piwi-expressing spermatogonial cells. **(B-B‴)** The *tsn* mutant testis. **(C-C‴)** The depletion of somatic Piwi did not rescue *tsn* mutant phenotype.
(TIF)

S12 Fig TSN shows little effects on piRNAs and transposon silencing in ovaries. (**A**) Small RNA composition in ovaries from the WT (left) and *tsn*^*1120*^*/tsn*^*0614*^ mutant (right) females. TE RNA, small RNAs derived from transposon regions; gene RNA, small RNAs derived from gene-coding regions; intergenic RNA, small RNAs derived from intergenic regions. (**B**) Size distribution of small RNAs (excluding miRNAs and fragments of long cellular RNAs such as tRNAs and rRNAs) in the WT (blue) and *tsn*^*1120*^*/tsn*^*0614*^ mutant (red) ovaries. The level of 25-29-nt small RNAs was slightly decreased in the *tsn* mutants. (**C**) Nucleotide composition of small RNAs (excluding miRNAs and fragments of long cellular RNAs such as tRNAs and rRNAs) in the WT (top) and *tsn*^*1120*^*/tsn*^*0614*^mutant (bottom) ovaries. The 1U-bias piRNA signature and nucleotide composition were not affected in *tsn* mutants. (**D**) Relative abundance of small RNAs derived from 19 previously reported piRNA clusters in *tsn* mutant ovaries compared to the WT ovaries are shown. Small RNAs uniquely mapped to these clusters were used for the analysis. These clusters are categorized into three groups according to the expression preference of their piRNAs (see references 39–41 in the main text), with the germline-enriched class colored in red, the intermediate class colored in blue, and the soma-enriched class colored in orange. No significant impact of *tsn* mutations on these piRNA clusters was observed. (**E**) qRT-PCR was performed to determine the expression of multiple transposons, relative to *rp49*, in *tsn*^*1120*^*/tsn*^*0614*^ mutant ovaries. Transcript levels from the WT ovaries were set as 1, and fold-changes are indicated. Error bars represent mean ± standard error of the mean (N = 3). *tsn* mutant ovaries displayed modest defects in transposon silencing, which is consistent with the data from testes (Fig 7E).
(TIF)

S13 Fig Papi and Tudor do not compensate for the loss of TSN in *tsn* mutant testes. Western blotting analysis of Papi and Tudor in *tsn* mutant and WT testes. No significant difference of Papi or Tudor expression was found between *tsn* mutant and *WT* control testes.
(TIF)

S14 Fig A model on the synergistic effect of TSN and Piwi in transposon silencing. TSN and Piwi act through independent pathways to silence transposons. In wildtype flies, the two pathways are both functional, which leads to complete silencing of transposons. In *piwi* mutants, a typical effect of transposon de-silencing is observed, as previously reported. In *tsn* mutants, a milder de-silencing effect is observed. This may not reflect that the TSN-mediated silencing has less function but rather because Piwi is significantly up-regulated in *tsn* mutants, which partially compensated for the loss of TSN-mediated transposon repression. In *tsn; piwi* double mutants, both TSN and Piwi-mediated repression mechanisms are abolished, which leads to an even more drastic transposon repression that has not been reported.
(TIF)

## References

[1] Juliano C, Wang J, Lin H. Uniting germline and stem cells: the function of Piwi proteins and the piRNA pathway in diverse organisms. Annual review of genetics. 2011;45:447–69. 10.1146/annurev-genet-110410-132541 21942366 PMC3832951

[2] Saxe JP, Lin H. Small noncoding RNAs in the germline. Cold Spring Harbor perspectives in biology. 2011;3(9):a002717 10.1101/cshperspect.a002717 21669983 PMC3181032

[3] Watanabe T, Cheng EC, Zhong M, Lin H. Retrotransposons and pseudogenes regulate mRNAs and lncRNAs via the piRNA pathway in the germline. Genome research. 2015;25(3):368–80. 10.1101/gr.180802.114 25480952 PMC4352877

[4] Peng JC, Lin H. Beyond transposons: the epigenetic and somatic functions of the Piwi-piRNA mechanism. Current opinion in cell biology. 2013;25(2):190–4. 10.1016/j.ceb.2013.01.010 23465540 PMC3651849

[5] Cox DN, Chao A, Lin H. piwi encodes a nucleoplasmic factor whose activity modulates the number and division rate of germline stem cells. Development. 2000;127(3):503–14. 10631171 10.1242/dev.127.3.503

[6] Cox DN, Chao A, Baker J, Chang L, Qiao D, Lin H. A novel class of evolutionarily conserved genes defined by piwi are essential for stem cell self-renewal. Genes & development. 1998;12(23):3715–27. 9851978 10.1101/gad.12.23.3715 PMC317255

[7] Megosh HB, Cox DN, Campbell C, Lin H. The role of PIWI and the miRNA machinery in Drosophila germline determination. Current biology: CB. 2006;16(19):1884–94. 16949822 10.1016/j.cub.2006.08.051

[8] Yin H, Lin H. An epigenetic activation role of Piwi and a Piwi-associated piRNA in Drosophila melanogaster. Nature. 2007;450(7167):304–8. Epub 2007/10/24. 17952056 10.1038/nature06263

[9] Callebaut I, Mornon JP. The human EBNA-2 coactivator p100: multidomain organization and relationship to the staphylococcal nuclease fold and to the tudor protein involved in Drosophila melanogaster development. Biochem J. 1997;321 (Pt 1):125–32. Epub 1997/01/01. 9003410 10.1042/bj3210125 PMC1218045

[10] Ponting CP. Tudor domains in proteins that interact with RNA. Trends Biochem Sci. 1997;22(2):51–2. Epub 1997/02/01. 9048482 10.1016/s0968-0004(96)30049-2

[11] Shaw N, Zhao M, Cheng C, Xu H, Saarikettu J, Li Y, et al The multifunctional human p100 protein 'hooks' methylated ligands. Nat Struct Mol Biol. 2007;14(8):779–84. Epub 2007/07/17. 17632523 10.1038/nsmb1269

[12] Fashe T, Saarikettu J, Isomaki P, Yang J, Silvennoinen O. Expression analysis of Tudor-SN protein in mouse tissues. Tissue Cell. 2013;45(1):21–31. Epub 2012/10/17. 10.1016/j.tice.2012.09.001 23068188

[13] Caudy AA, Ketting RF, Hammond SM, Denli AM, Bathoorn AM, Tops BB, et al A micrococcal nuclease homologue in RNAi effector complexes. Nature. 2003;425(6956):411–4. Epub 2003/09/26. 14508492 10.1038/nature01956

[14] Li CL, Yang WZ, Chen YP, Yuan HS. Structural and functional insights into human Tudor-SN, a key component linking RNA interference and editing. Nucleic Acids Research. 2008;36(11):3579–89. 10.1093/nar/gkn236 18453631 PMC2441809

[15] Gao X, Zhao X, Zhu Y, He J, Shao J, Su C, et al Tudor staphylococcal nuclease (Tudor-SN) participates in small ribonucleoprotein (snRNP) assembly via interacting with symmetrically dimethylated Sm proteins. J Biol Chem. 2012;287(22):18130–41. Epub 2012/04/12. 10.1074/jbc.M111.311852 22493508 PMC3365748

[16] Gao X, Ge L, Shao J, Su C, Zhao H, Saarikettu J, et al Tudor-SN interacts with and co-localizes with G3BP in stress granules under stress conditions. FEBS Lett. 2010;584(16):3525–32. Epub 2010/07/21. 10.1016/j.febslet.2010.07.022 20643132 PMC7127458

[17] Scadden AD. The RISC subunit Tudor-SN binds to hyper-edited double-stranded RNA and promotes its cleavage. Nat Struct Mol Biol. 2005;12(6):489–96. Epub 2005/05/17. 15895094 10.1038/nsmb936

[18] Yang J, Aittomaki S, Pesu M, Carter K, Saarinen J, Kalkkinen N, et al Identification of p100 as a coactivator for STAT6 that bridges STAT6 with RNA polymerase II. EMBO J. 2002;21(18):4950–8. Epub 2002/09/18. 12234934 10.1093/emboj/cdf463 PMC126276

[19] Paukku K, Yang J, Silvennoinen O. Tudor and nuclease-like domains containing protein p100 function as coactivators for signal transducer and activator of transcription 5. Mol Endocrinol. 2003;17(9):1805–14. Epub 2003/06/24. 12819296 10.1210/me.2002-0256

[20] Liu K, Chen C, Guo Y, Lam R, Bian C, Xu C, et al Structural basis for recognition of arginine methylated Piwi proteins by the extended Tudor domain. Proc Natl Acad Sci U S A. 2010;107(43):18398–403. Epub 2010/10/13. 10.1073/pnas.1013106107 20937909 PMC2972943

[21] Vagin VV, Wohlschlegel J, Qu J, Jonsson Z, Huang X, Chuma S, et al Proteomic analysis of murine Piwi proteins reveals a role for arginine methylation in specifying interaction with Tudor family members. Genes Dev. 2009;23(15):1749–62. Epub 2009/07/09. 10.1101/gad.1814809 19584108 PMC2720255

[22] Liu L, Qi H, Wang J, Lin H. PAPI, a novel TUDOR-domain protein, complexes with AGO3, ME31B and TRAL in the nuage to silence transposition. Development. 2011;138(9):1863–73. 10.1242/dev.059287 21447556 PMC3074456

[23] Gangaraju VK, Yin H, Weiner MM, Wang JQ, Huang XA, Lin HF. Drosophila Piwi functions in Hsp90-mediated suppression of phenotypic variation. Nature Genetics. 2011;43(2):153–U07. 10.1038/ng.743 21186352 PMC3443399

[24] Cooper JL, Till BJ, Henikoff S. Fly-TILL: reverse genetics using a living point mutation resource. Fly (Austin). 2008;2(6):300–2. Epub 2008/12/23. 19098435 10.4161/fly.7366

[25] Lin H, Yue L, Spradling AC. The Drosophila fusome, a germline-specific organelle, contains membrane skeletal proteins and functions in cyst formation. Development. 1994;120(4):947–56. Epub 1994/04/01. 7600970 10.1242/dev.120.4.947

[26] Yang W, Chendrimada TP, Wang Q, Higuchi M, Seeburg PH, Shiekhattar R, et al Modulation of microRNA processing and expression through RNA editing by ADAR deaminases. Nat Struct Mol Biol. 2006;13(1):13–21. Epub 2005/12/22. 16369484 10.1038/nsmb1041 PMC2950615

[27] Cappellari M, Bielli P, Paronetto MP, Ciccosanti F, Fimia GM, Saarikettu J, et al The transcriptional co-activator SND1 is a novel regulator of alternative splicing in prostate cancer cells. Oncogene. 2013. Epub 2013/09/03. 10.1038/onc.2013.360 23995791

[28] Blanco MA, Aleckovic M, Hua Y, Li T, Wei Y, Xu Z, et al Identification of staphylococcal nuclease domain-containing 1 (SND1) as a Metadherin-interacting protein with metastasis-promoting functions. J Biol Chem. 2011;286(22):19982–92. Epub 2011/04/12. 10.1074/jbc.M111.240077 21478147 PMC3103372

[29] Kuruma H, Kamata Y, Takahashi H, Igarashi K, Kimura T, Miki K, et al Staphylococcal nuclease domain-containing protein 1 as a potential tissue marker for prostate cancer. Am J Pathol. 2009;174(6):2044–50. Epub 2009/05/14. 10.2353/ajpath.2009.080776 19435788 PMC2684170

[30] Tsuchiya N, Ochiai M, Nakashima K, Ubagai T, Sugimura T, Nakagama H. SND1, a component of RNA-induced silencing complex, is up-regulated in human colon cancers and implicated in early stage colon carcinogenesis. Cancer Res. 2007;67(19):9568–76. Epub 2007/10/03. 17909068 10.1158/0008-5472.CAN-06-2707

[31] Janic A, Mendizabal L, Llamazares S, Rossell D, Gonzalez C. Ectopic expression of germline genes drives malignant brain tumor growth in Drosophila. Science. 2010;330(6012):1824–7. Epub 2011/01/06. 10.1126/science.1195481 21205669

[32] Qiao D, Zeeman AM, Deng W, Looijenga LH, Lin H. Molecular characterization of hiwi, a human member of the piwi gene family whose overexpression is correlated to seminomas. Oncogene. 2002;21(25):3988–99. 12037681 10.1038/sj.onc.1205505

[33] Ross RJ, Weiner MM, Lin H. PIWI proteins and PIWI-interacting RNAs in the soma. Nature. 2014;505(7483):353–9. 10.1038/nature12987 24429634 PMC4265809

[34] Saito K, Nishida KM, Mori T, Kawamura Y, Miyoshi K, Nagami T, et al Specific association of Piwi with rasiRNAs derived from retrotransposon and heterochromatic regions in the Drosophila genome. Genes Dev. 2006;20(16):2214–22. Epub 2006/08/03. 16882972 10.1101/gad.1454806 PMC1553205

[35] Saarikettu J, Ovod V, Vuoksio M, Gronholm J, Yang J, Silvennoinen O. Monoclonal antibodies against human Tudor-SN. Hybridoma (Larchmt). 2010;29(3):231–6. Epub 2010/06/24. 20568998 10.1089/hyb.2009.0114

[36] Lin H, Spradling AC. A novel group of pumilio mutations affects the asymmetric division of germline stem cells in the Drosophila ovary. Development. 1997;124(12):2463–76. Epub 1997/06/01. 9199372 10.1242/dev.124.12.2463

[37] Jurka J, Kapitonov VV, Pavlicek A, Klonowski P, Kohany O, Walichiewicz J. Repbase Update, a database of eukaryotic repetitive elements. Cytogenet Genome Res. 2005;110(1–4):462–7. Epub 2005/08/12. 16093699 10.1159/000084979

[38] Qi H, Watanabe T, Ku HY, Liu N, Zhong M, Lin H. The Yb Body, a Major Site for Piwi-associated RNA Biogenesis and a Gateway for Piwi Expression and Transport to the Nucleus in Somatic Cells. Journal of Biological Chemistry. 2010;286(5):3789–97. 10.1074/jbc.M110.193888 21106531 PMC3030380

[39] Saito K, Inagaki S, Mituyama T, Kawamura Y, Ono Y, Sakota E, et al A regulatory circuit for piwi by the large Maf gene traffic jam in Drosophila. Nature. 2009;461(7268):1296–9. Epub 2009/10/09. 10.1038/nature08501 19812547

[40] Malone CD, Brennecke J, Dus M, Stark A, McCombie WR, Sachidanandam R, et al Specialized piRNA pathways act in germline and somatic tissues of the Drosophila ovary. Cell. 2009;137(3):522–35. Epub 2009/04/28. 10.1016/j.cell.2009.03.040 19395010 PMC2882632

[41] Lau NC, Robine N, Martin R, Chung WJ, Niki Y, Berezikov E, et al Abundant primary piRNAs, endo-siRNAs, and microRNAs in a Drosophila ovary cell line. Genome Res. 2009;19(10):1776–85. Epub 2009/06/23. 10.1101/gr.094896.109 19541914 PMC2765285

