# Supplementary figures and images for: Tudor-SN Interacts with Piwi Antagonistically in Regulating Spermatogenesis but Synergistically in Silencing Transposons in Drosophila
Source: PLoS Genet. 2016 Jan 25;12(1):e1005813. doi: 10.1371/journal.pgen.1005813 (PMC4726654; doi:10.1371/journal.pgen.1005813)

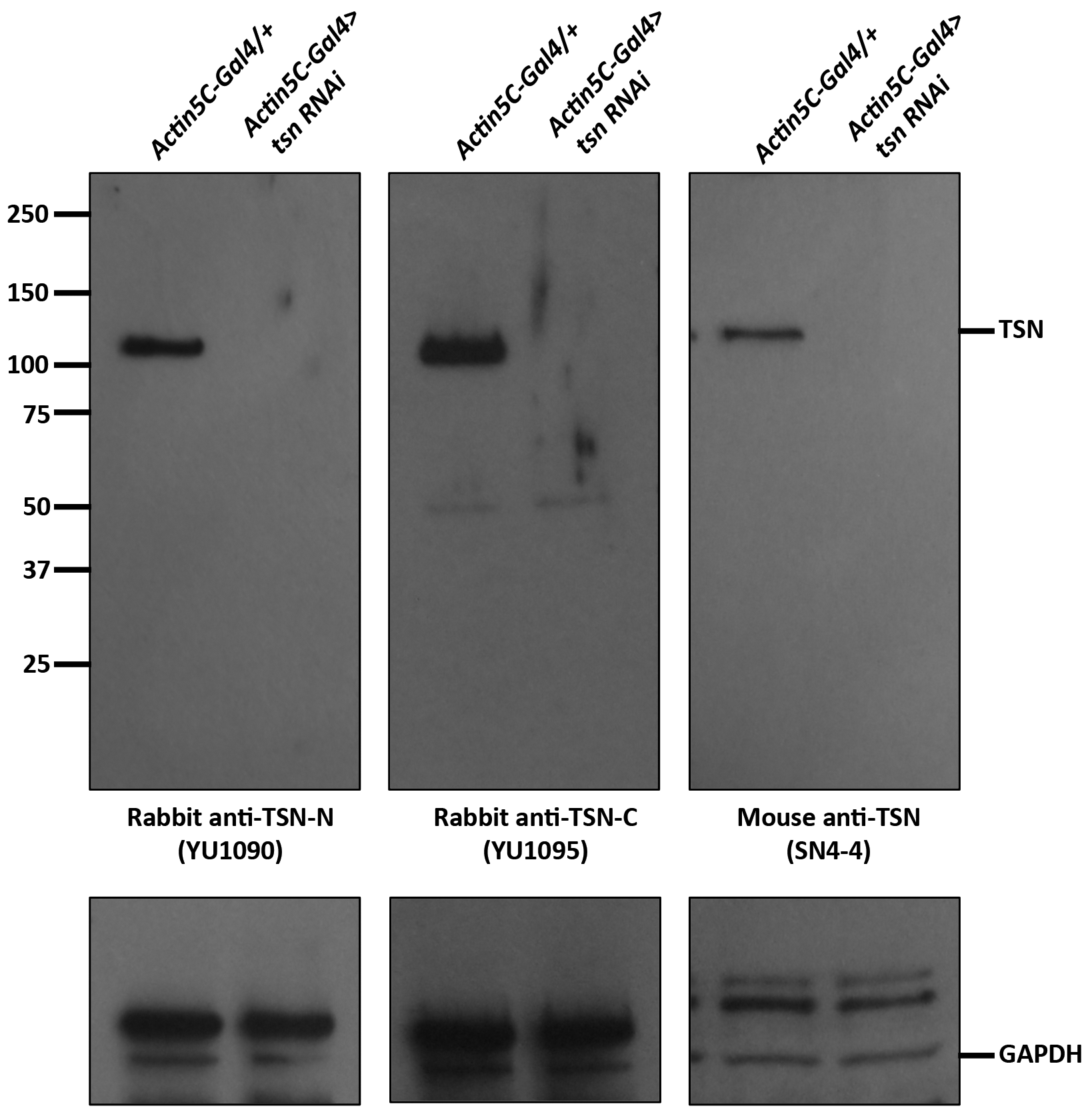

Supplement: S1 Fig — Western blot analysis using the ovarian lysates prepared from tsn knockdown and control females. The blots were probed with polyclonal rabbit anti-TSN-N (left), polyclonal rabbit anti-TSN-C (middle), and monoclonal mouse anti-TSN (right) antibodies. TSN is ~100 kDa. (TIF) [file pgen.1005813.s001.tif]

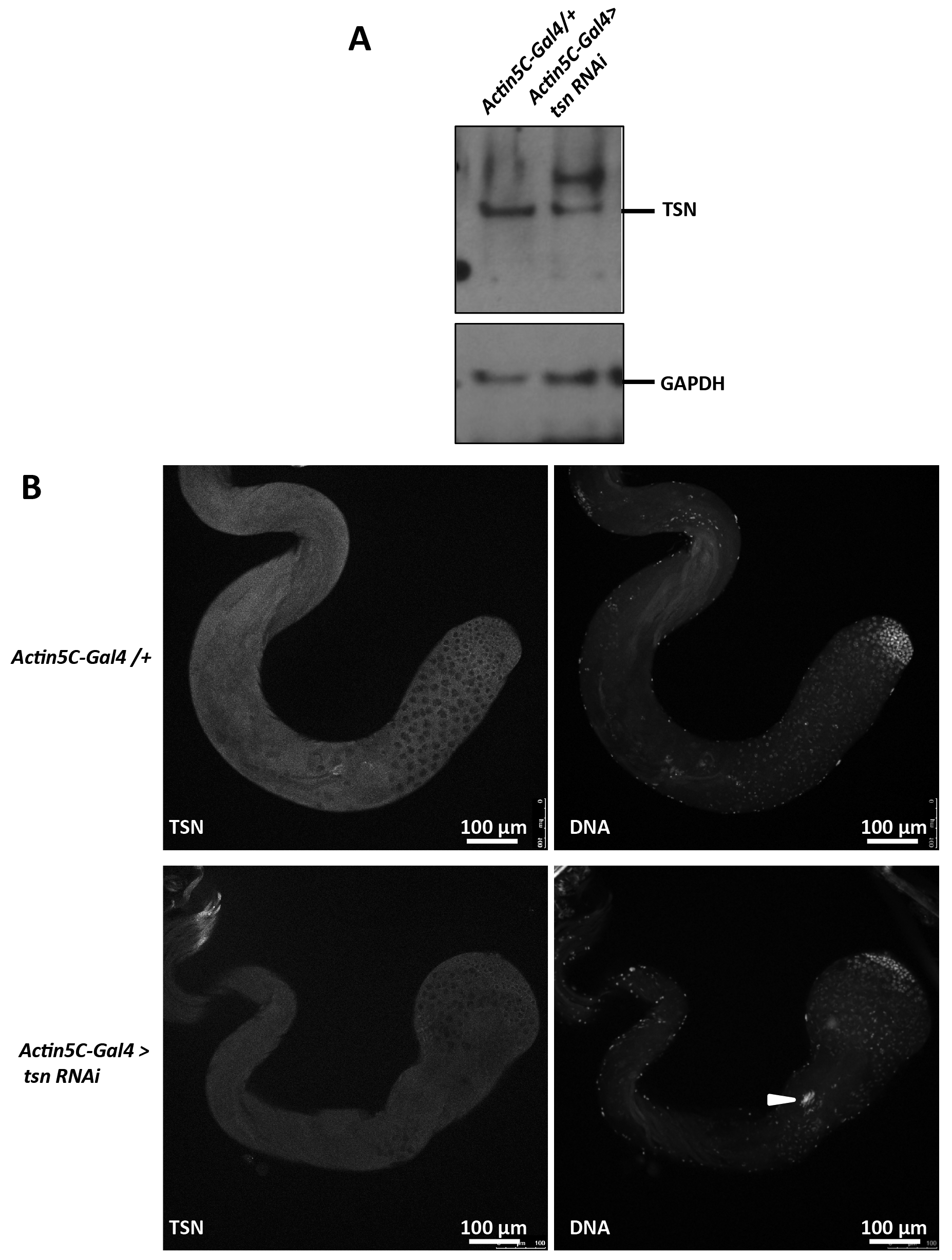

Supplement: S2 Fig — (A) Western blot analysis using mouse anti-TSN antibody showing the knockdown efficiency of tsn in testes. (B) Testes from tsn knockdown and control males were immunostained with mouse anti-TSN antibody. DNA was labeled by DAPI (blue). The knockdown of tsn caused the swollen apical tip of the testis (see text). Arrow indicates a bundle of elongated spermatids with their heads resided at the apical tip region of the testis. (TIF) [file pgen.1005813.s002.tif]

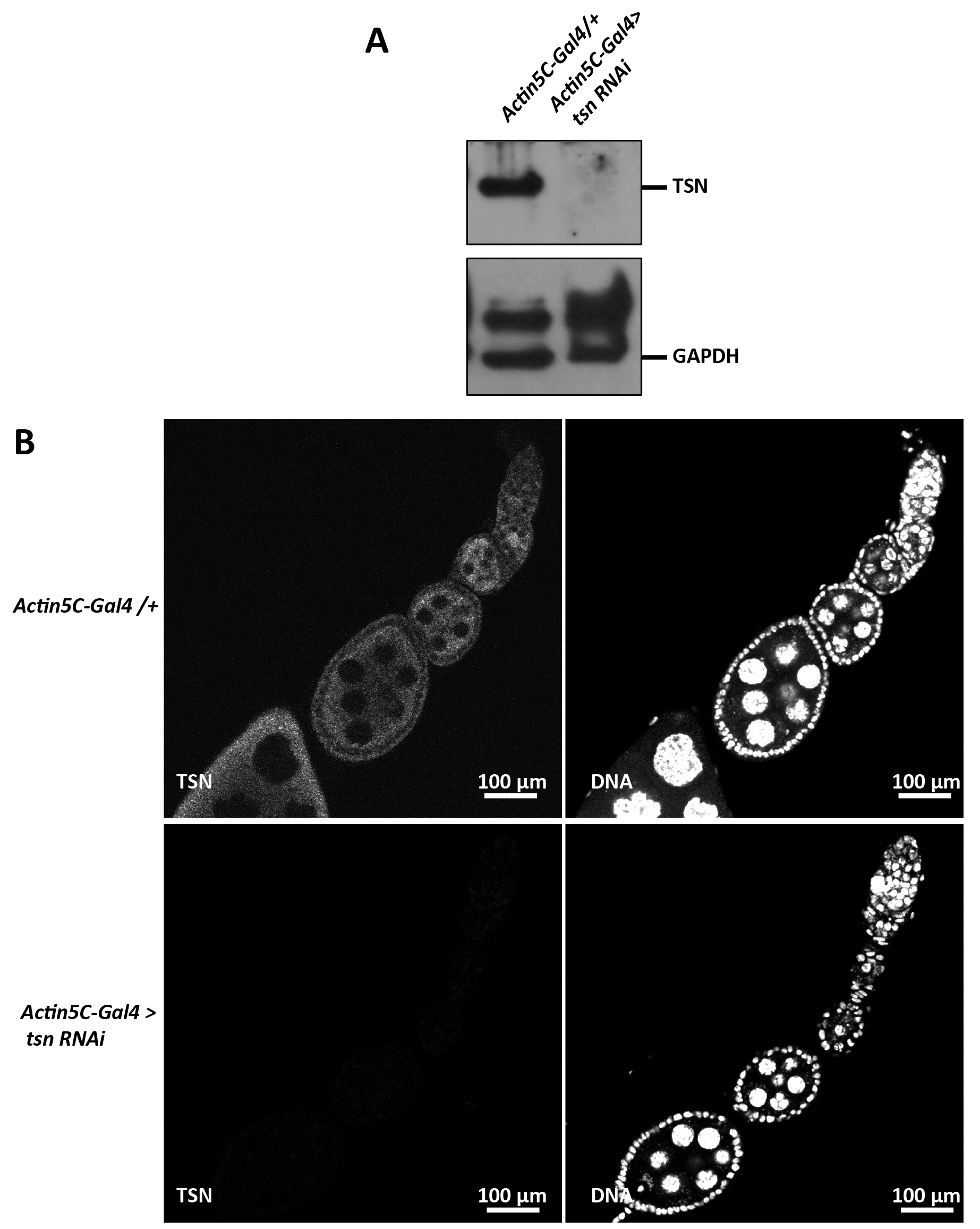

Supplement: S3 Fig — (A) Western blot analysis using mouse anti-TSN antibody showing the knockdown efficiency of tsn in ovaries. (B) Ovaries from tsn knockdown and control females were immunostained with mouse anti-TSN antibody. DNA was labeled by DAPI (blue). The knockdown of tsn did not generate any obvious defects in oogenesis. (TIF) [file pgen.1005813.s003.tif]

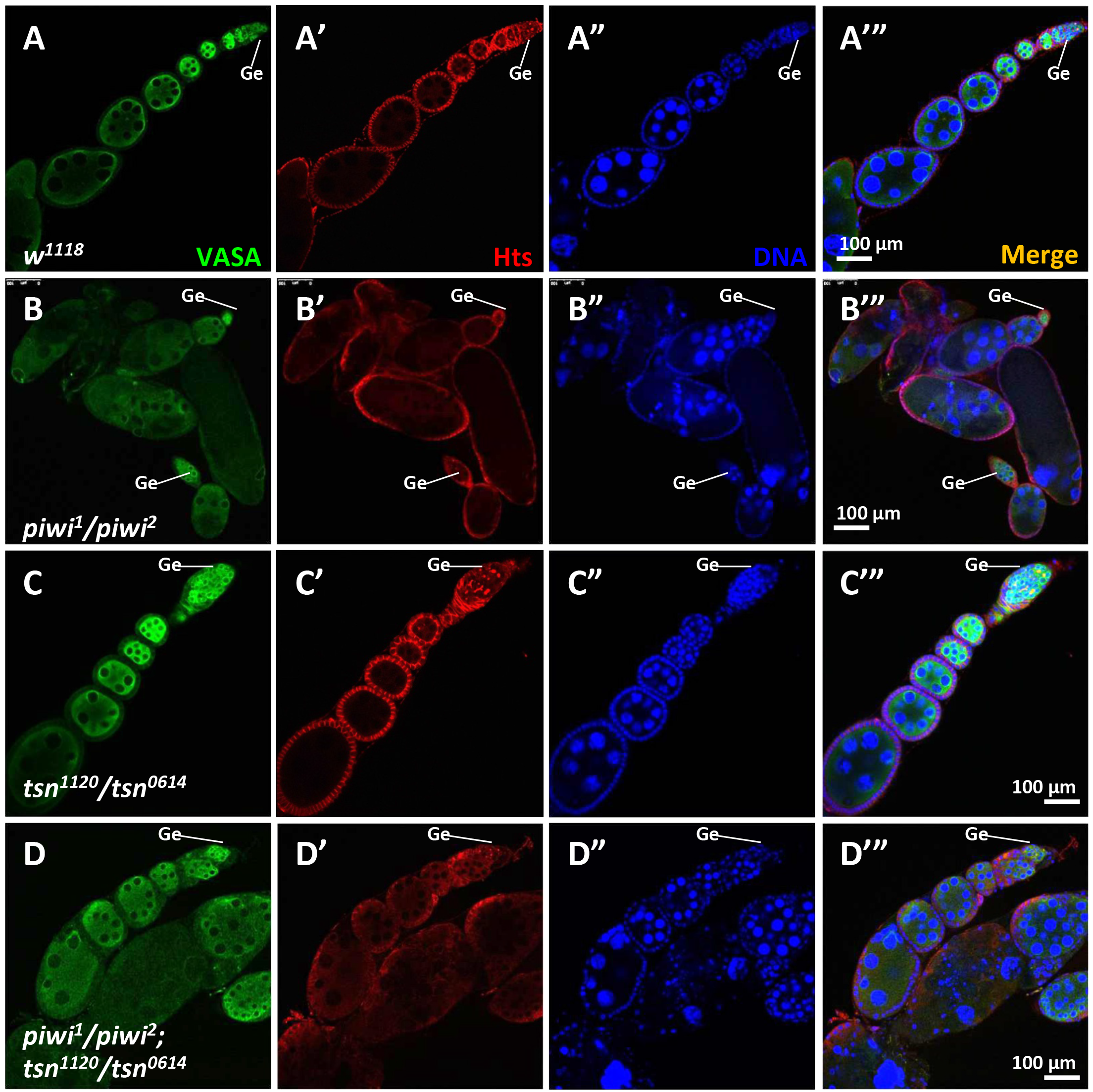

Supplement: S4 Fig — Immunostaining of VASA (green) and Hts (red) and in ovaries from WT (A-A‴), piwi mutants (B-B‴), tsn mutants (C-C‴), and piwi and tsn double mutants (D-D‴). The defect of piwi mutant ovaries (B-B‴) was significantly rescued by tsn mutations (D-D‴). This antagonistic relationship between TSN and PIWI in oogenesis parallels that in spermatogensis (Fig 4A–4D). (TIF) [file pgen.1005813.s004.tif]

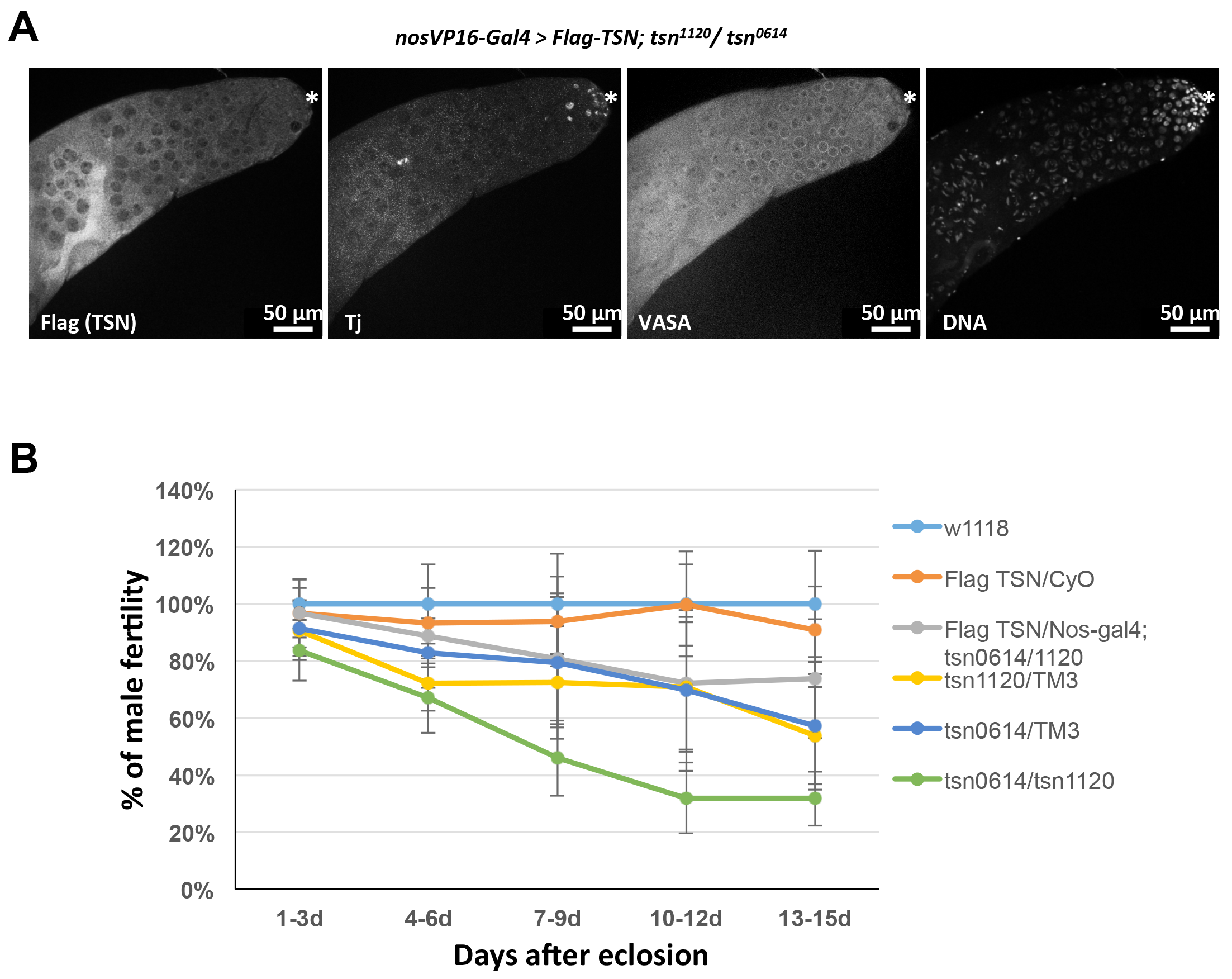

Supplement: S5 Fig — (A). Flag-TSN was expressed in germ cells by nosVP16-Gal4 in the tsn1120/tsn0614 mutant background. The testes were immunostained with anti-Flag (Flag-TSN), anti-Tj (somatic cells), and anti-VASA (germ cells) antibodies. DNA was labeled by DAPI (blue). Asterisk indicates the hub. tsn mutant phenotype was rescued by the expression of full-length WT TSN. (B). Fertility assay of piwi and tsn double mutant males. The reduced fertility of tsn mutant males (red line) was restored largely by expression of Flag-tagged TSN in the germline cells driven by VP16-NosGal4 driver at the tsn mutant background (gray line). The fertility of WT males is shown as the light blue line. (TIF) [file pgen.1005813.s005.tif]

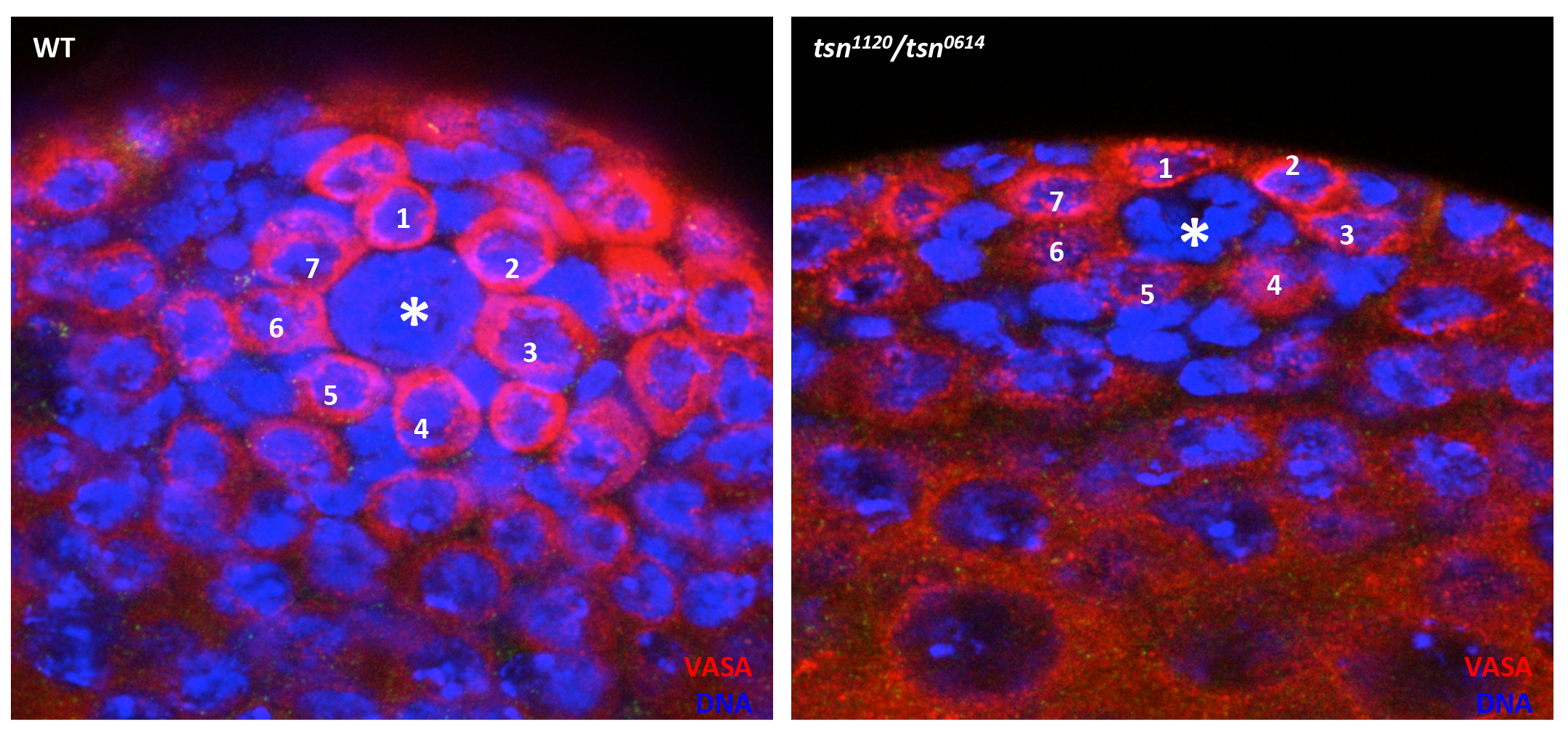

Supplement: S6 Fig — Germ cells were labeled with anti-VASA antibody (red) and DNA was labeled by DAPI (blue). The average numbers of GSCs in WT and tsn mutant testes was 6.67 (n = 12) and 6.5 (n = 12), respectively. There were no obvious abnormalities in GSCs of tsn mutant testes, suggesting the phenotype of tsn mutants may not be due to defects in GSCs. (TIF) [file pgen.1005813.s006.tif]

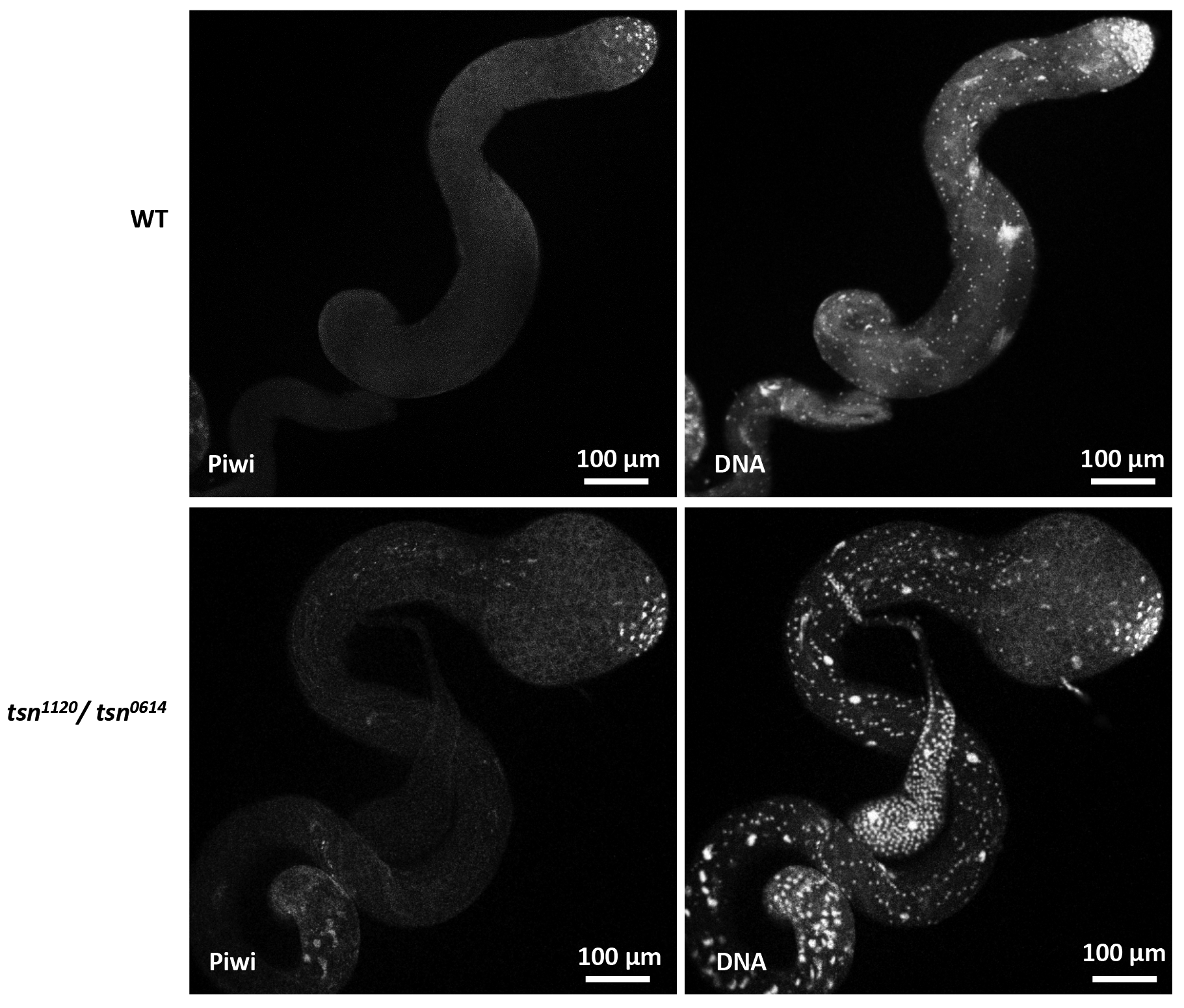

Supplement: S7 Fig — Immunostaining of Piwi in testes from adult WT and tsn mutant males. DNA was labeled by DAPI. Piwi was expressed in early germ cells and somatic cyst cells in both WT and tsn mutant testes. This result suggests the upregulation of Piwi is in the Piwi-expressing cells, but not caused by ectopic expression of Piwi. (TIF) [file pgen.1005813.s007.tif]

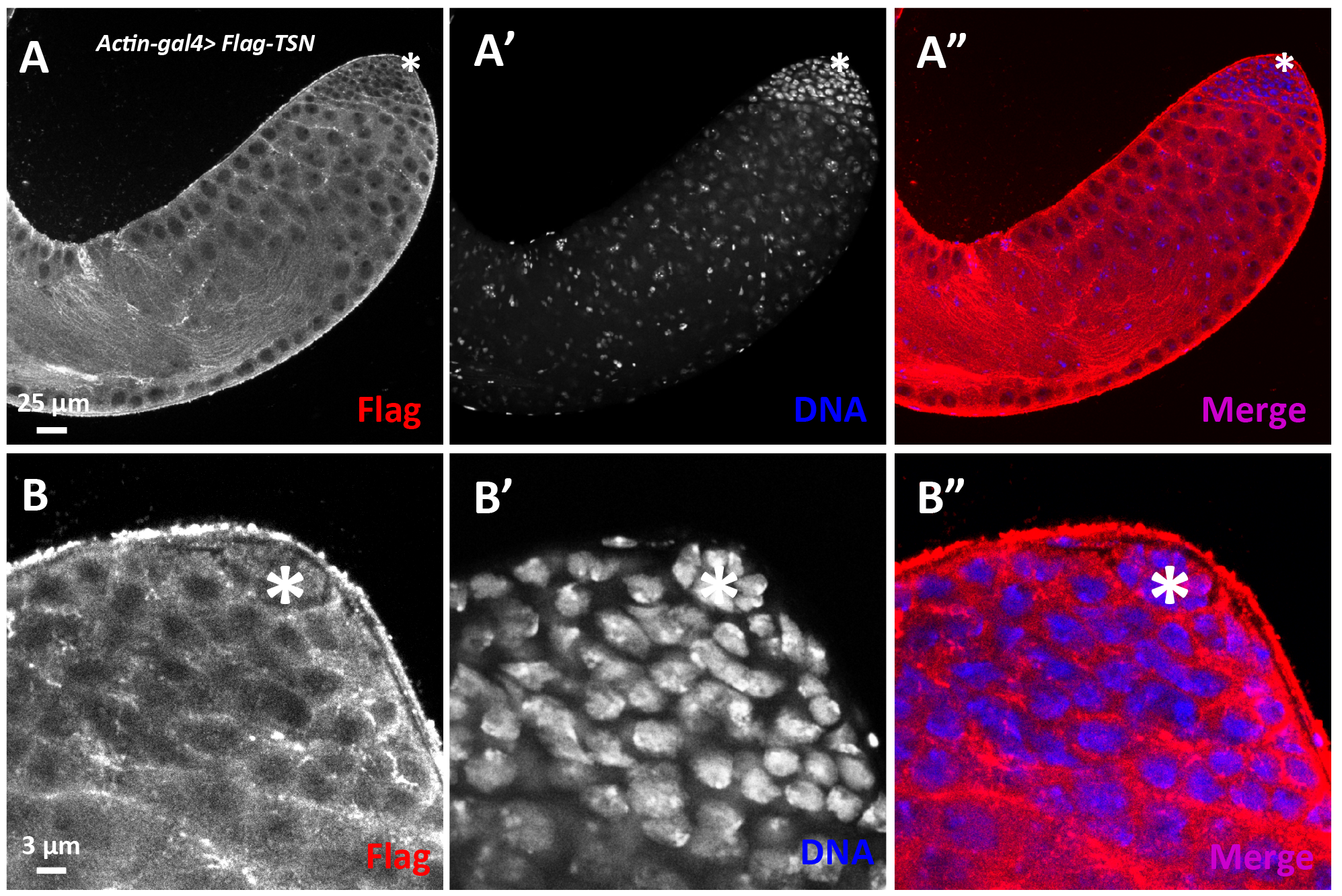

Supplement: S8 Fig — Germline-specific expression of the Flag-tagged tsn transgene was induced by nosVP16-Gal4 in w1118 male flies. (A, B) Testes from 2-day-old males were immunostained with mouse anti-Flag M2 antibody. (A’, B’) DNA was labeled by DAPI (blue). (A”, B”) merged images for Flag and DAPI staining. Asterisks indicate the hubs. Overexpression of tsn in WT germ cells does not cause any obvious phenotype. (TIF) [file pgen.1005813.s008.tif]

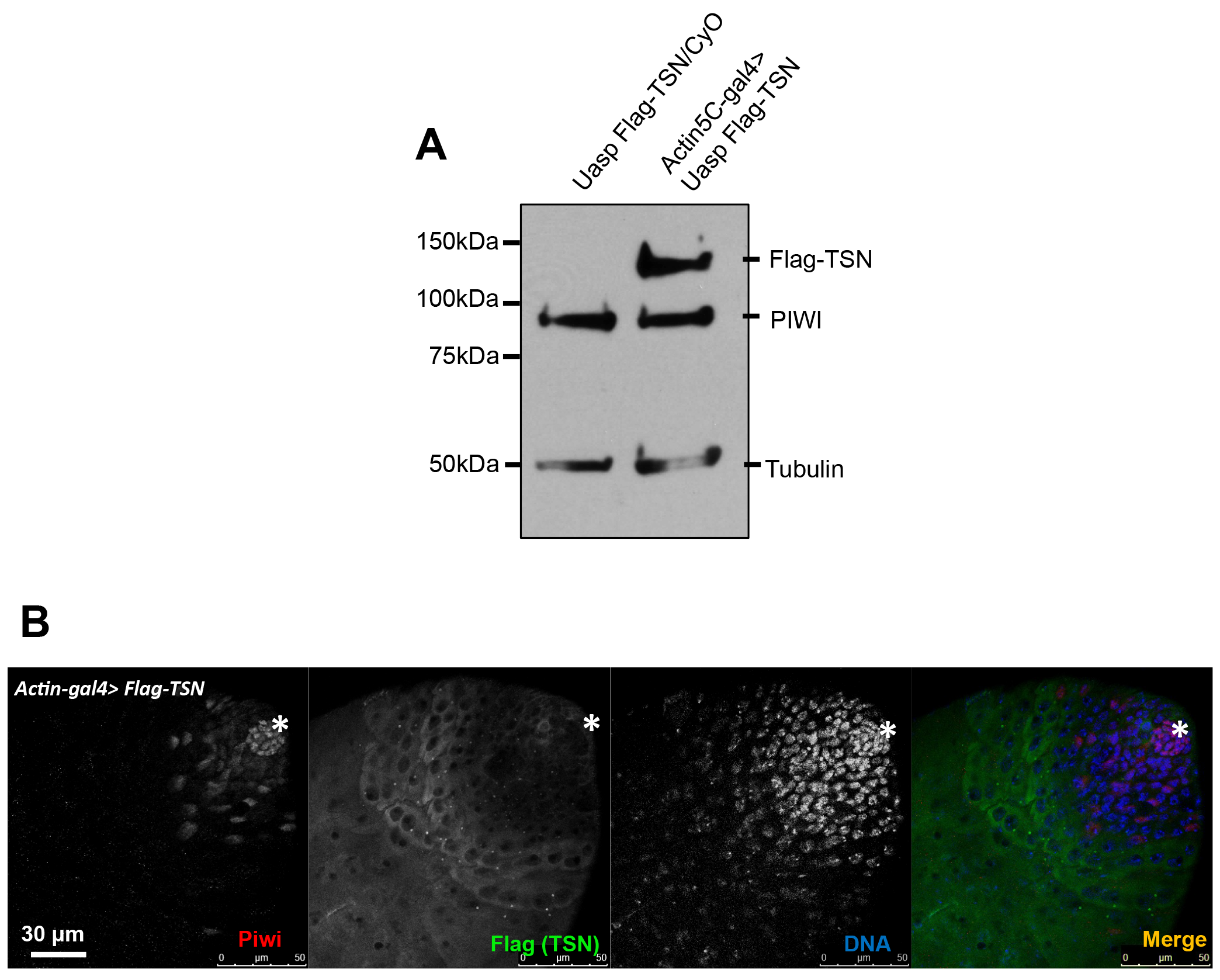

Supplement: S9 Fig — Overexpression of Flag-tagged TSN was induced by Actin5C-Gal4 in w1118 male flies. 2-day-old testes were used for the analysis. (A) Western analysis of TSN and Piwi. Overexpressed Flag-TSN was examined with mouse anti-Flag M2 antibody. Piwi expression level is slightly reduced in TSN overexpressed testes comparing to WT testes. (B) Immunostaining of Flag-TSN and Piwi in Flag-TSN overexpressing testes. DNA was labeled by DAPI (blue). Asterisks indicate the hubs. Piwi localization is normal while its expression level especially in somatic cyst cells is reduced. (TIF) [file pgen.1005813.s009.tif]

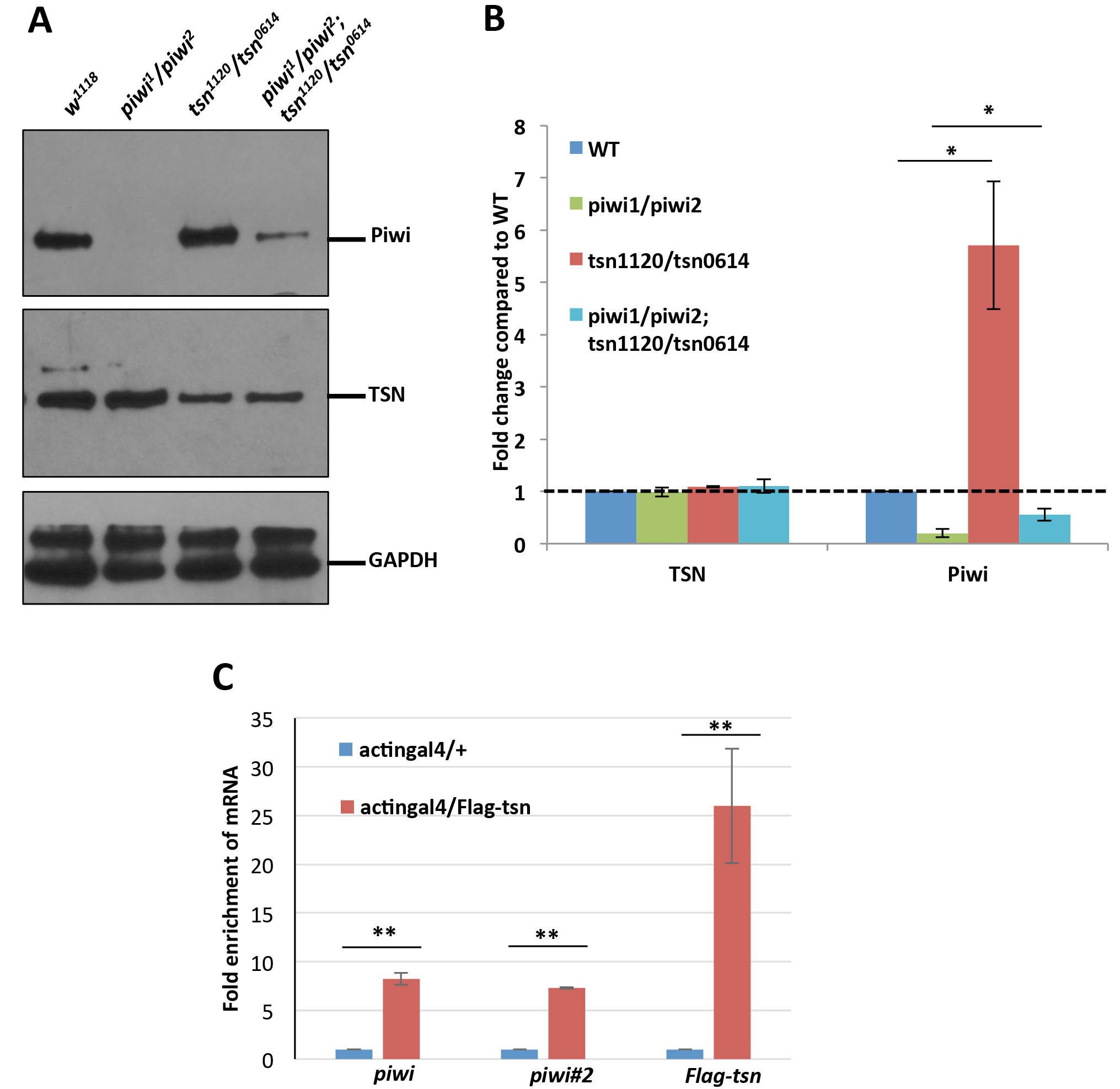

Supplement: S10 Fig — (A) Western blot analysis of Piwi expression level in piwi mutant, tsn mutant, and piwi and tsn double mutant ovaries. Piwi protein level was upregulated in tsn mutants, suggesting a negative regulation of TSN on Piwi expression. (B) qRT-PCR of Piwi and TSN in piwi mutant, tsn mutant, and piwi and tsn double mutant ovaries. Piwi mRNA level was upregulated in tsn mutant ovaries, indicating TSN negatively regulates Piwi expression at the mRNA level. (C). TSN binds to piwi mRNA in Drosophila ovaries. qRT-PCR to detect piwi mRNA (using two sets of primers: piwi and piwi#2) and tsn mRNA from RNA co-immunoprecipitated from wildtype and Flag-TSN overexpressing ovaries using anti-Flag antibody. Error bars represent mean ± standard error of the mean (N ≧ 3). *P < 0.05 and **P<0.01. (TIF) [file pgen.1005813.s010.tif]

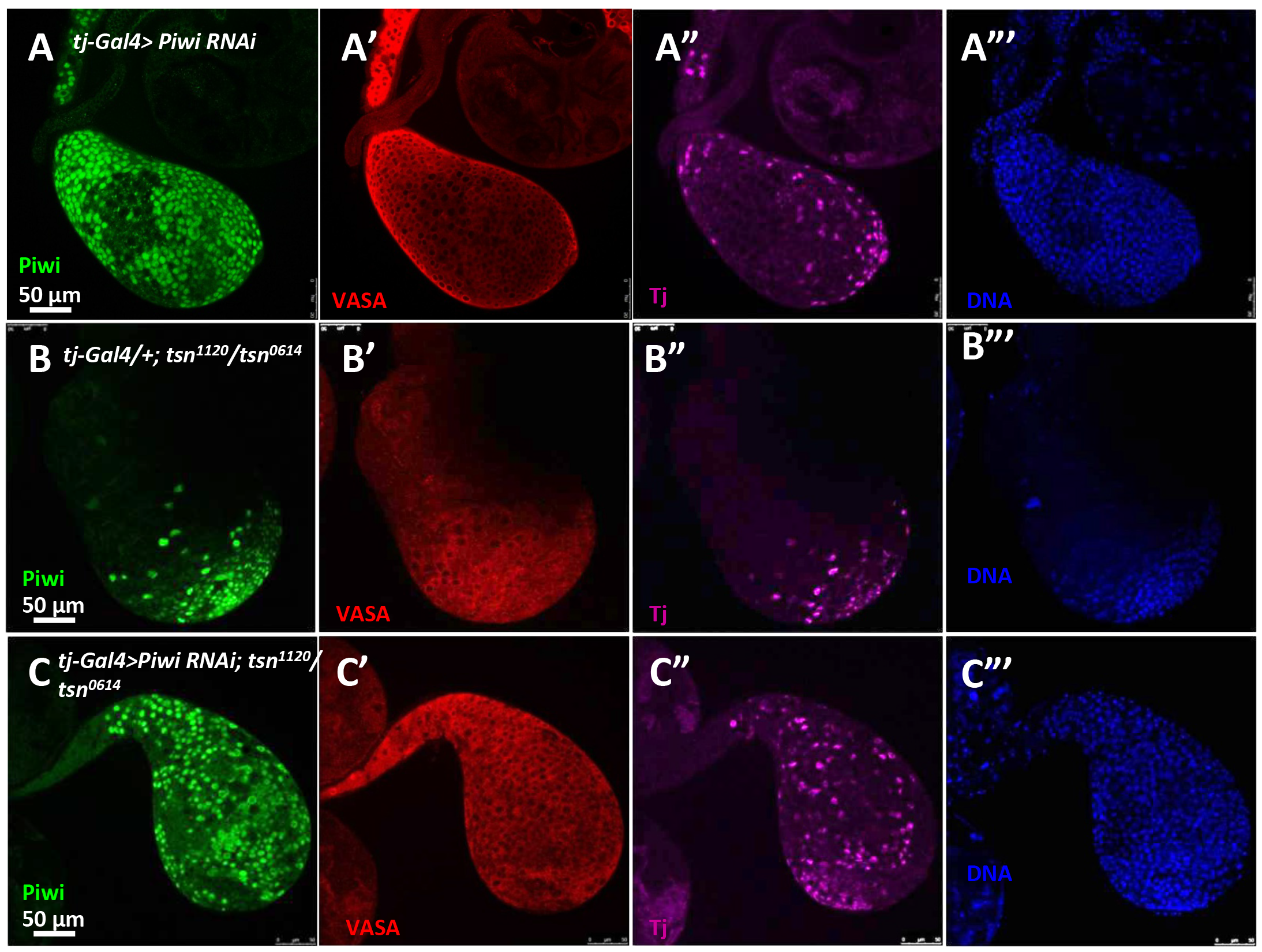

Supplement: S11 Fig — The testes were immunostained with anti-Piwi (green), anti-VASA (germ cell, red), and anti-Tj (somatic cells, purple) antibodies. DNA was labeled by DAPI (blue). (A-A‴) Somatic Piwi was knockdown using tj-Gal4 driver. The knockdown of somatic Piwi resulted in an expansion of Piwi-expressing spermatogonial cells. (B-B‴) The tsn mutant testis. (C-C‴) The depletion of somatic Piwi did not rescue tsn mutant phenotype. (TIF) [file pgen.1005813.s011.tif]

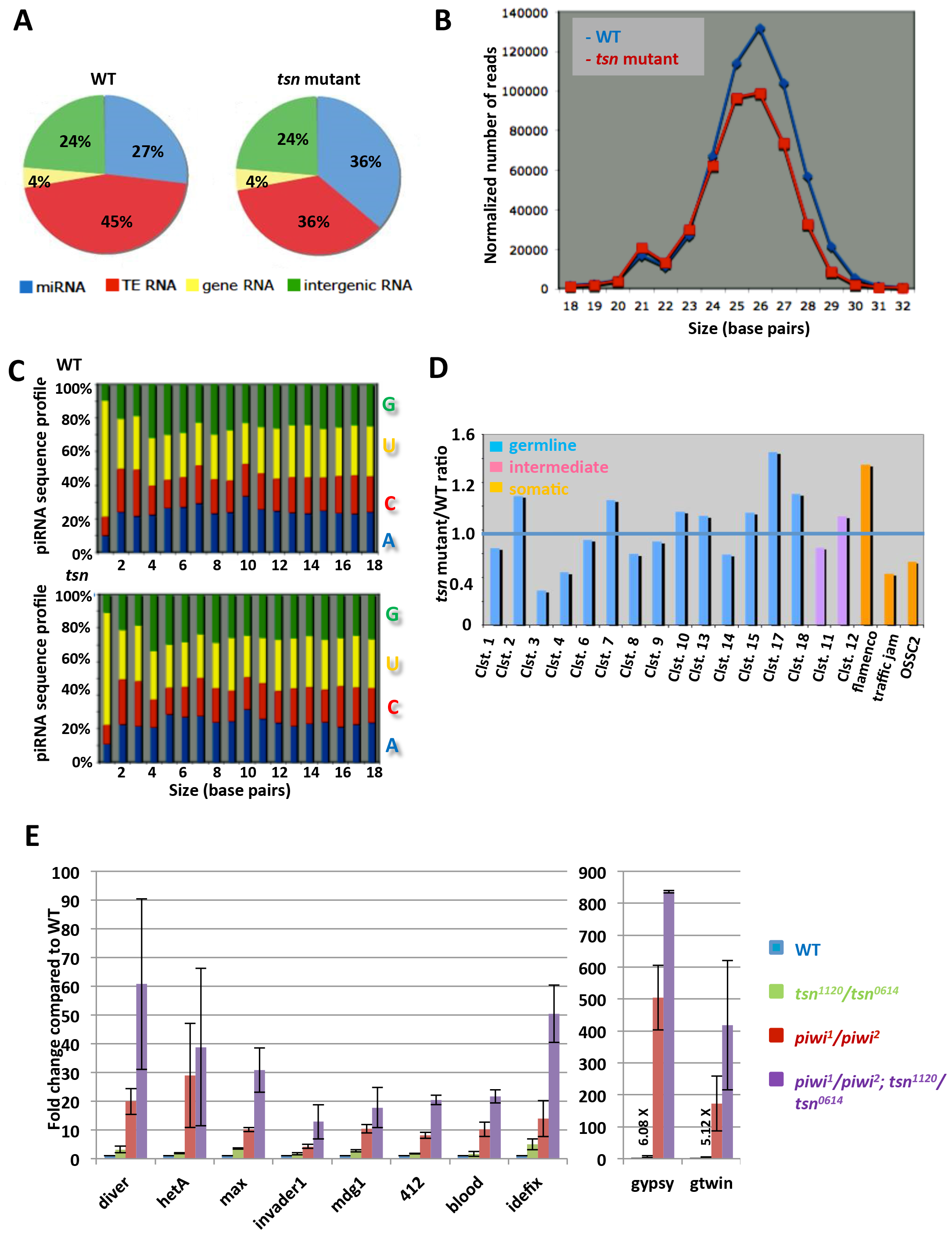

Supplement: S12 Fig — (A) Small RNA composition in ovaries from the WT (left) and tsn1120/tsn0614 mutant (right) females. TE RNA, small RNAs derived from transposon regions; gene RNA, small RNAs derived from gene-coding regions; intergenic RNA, small RNAs derived from intergenic regions. (B) Size distribution of small RNAs (excluding miRNAs and fragments of long cellular RNAs such as tRNAs and rRNAs) in the WT (blue) and tsn1120/tsn0614 mutant (red) ovaries. The level of 25-29-nt small RNAs was slightly decreased in the tsn mutants. (C) Nucleotide composition of small RNAs (excluding miRNAs and fragments of long cellular RNAs such as tRNAs and rRNAs) in the WT (top) and tsn1120/tsn0614mutant (bottom) ovaries. The 1U-bias piRNA signature and nucleotide composition were not affected in tsn mutants. (D) Relative abundance of small RNAs derived from 19 previously reported piRNA clusters in tsn mutant ovaries compared to the WT ovaries are shown. Small RNAs uniquely mapped to these clusters were used for the analysis. These clusters are categorized into three groups according to the expression preference of their piRNAs (see references 39–41 in the main text), with the germline-enriched class colored in red, the intermediate class colored in blue, and the soma-enriched class colored in orange. No significant impact of tsn mutations on these piRNA clusters was observed. (E) qRT-PCR was performed to determine the expression of multiple transposons, relative to rp49, in tsn1120/tsn0614 mutant ovaries. Transcript levels from the WT ovaries were set as 1, and fold-changes are indicated. Error bars represent mean ± standard error of the mean (N = 3). tsn mutant ovaries displayed modest defects in transposon silencing, which is consistent with the data from testes (Fig 7E). (TIF) [file pgen.1005813.s012.tif]

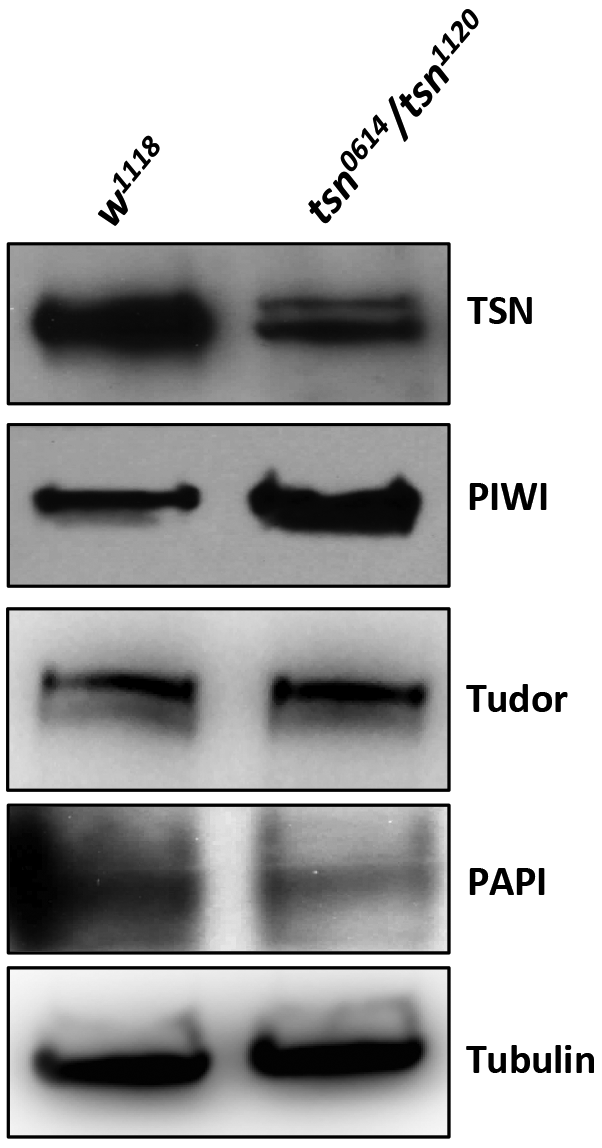

Supplement: S13 Fig — Western blotting analysis of Papi and Tudor in tsn mutant and WT testes. No significant difference of Papi or Tudor expression was found between tsn mutant and WT control testes. (TIF) [file pgen.1005813.s013.tif]

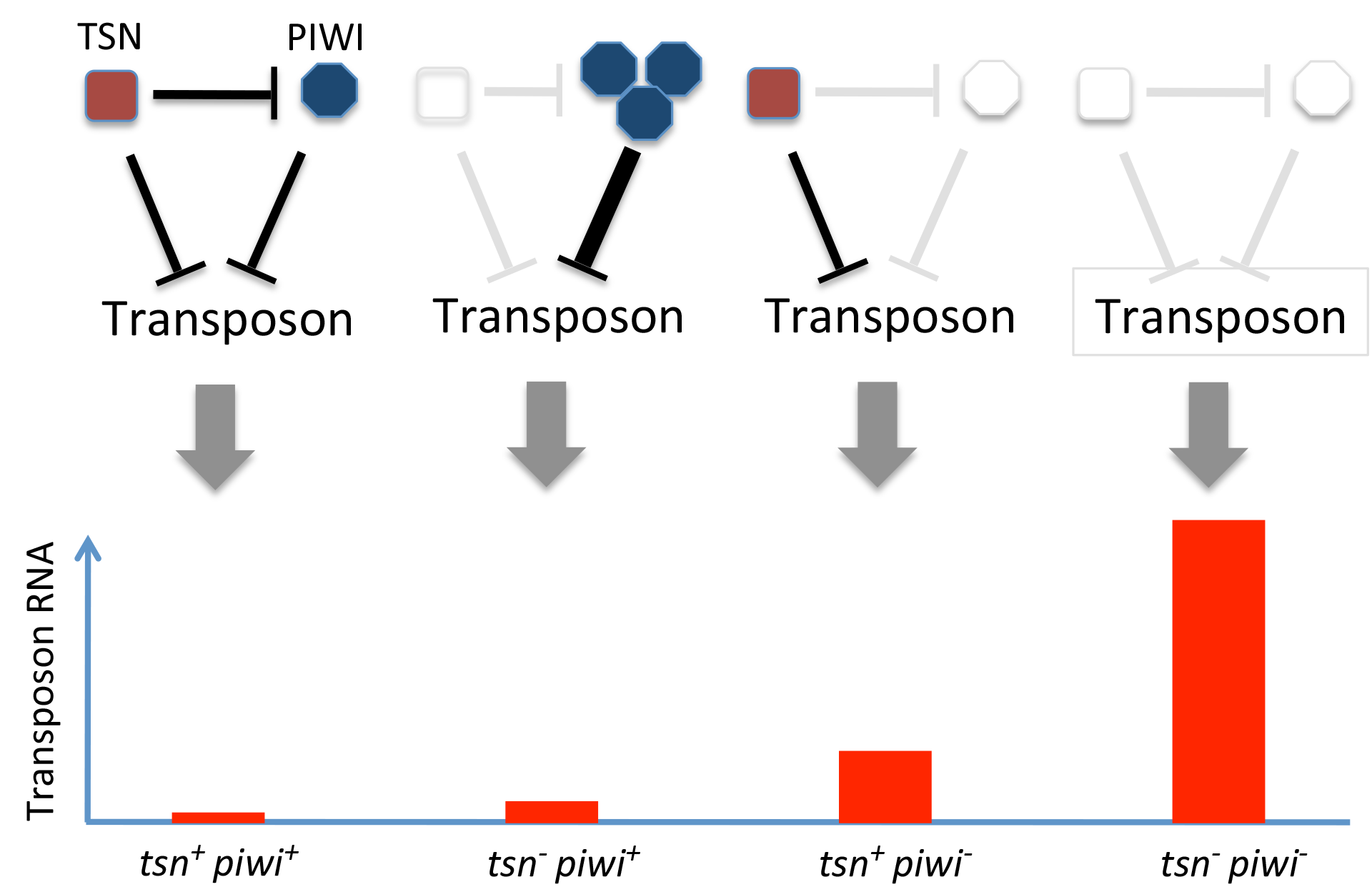

Supplement: S14 Fig — TSN and Piwi act through independent pathways to silence transposons. In wildtype flies, the two pathways are both functional, which leads to complete silencing of transposons. In piwi mutants, a typical effect of transposon de-silencing is observed, as previously reported. In tsn mutants, a milder de-silencing effect is observed. This may not reflect that the TSN-mediated silencing has less function but rather because Piwi is significantly up-regulated in tsn mutants, which partially compensated for the loss of TSN-mediated transposon repression. In tsn; piwi double mutants, both TSN and Piwi-mediated repression mechanisms are abolished, which leads to an even more drastic transposon repression that has not been reported. (TIF) [file pgen.1005813.s014.tif]
